# De novo reconstruction of cell interaction landscapes from single-cell spatial transcriptome data with DeepLinc

**DOI:** 10.1186/s13059-022-02692-0

**Published:** 2022-06-03

**Authors:** Runze Li, Xuerui Yang

**Affiliations:** MOE Key Laboratory of Bioinformatics, Center for Synthetic & Systems Biology, School of Life Sciences, Tsinghua University, Beijing, 100084 China

**Keywords:** Single-cell spatial transcriptome, Cell interaction, Variational graph autoencoder, VGAE, Deep learning

## Abstract

Based on a deep generative model of variational graph autoencoder (VGAE), we develop a new method, DeepLinc (deep learning framework for Landscapes of Interacting Cells), for the de novo reconstruction of cell interaction networks from single-cell spatial transcriptomic data. DeepLinc demonstrates high efficiency in learning from imperfect and incomplete spatial transcriptome data, filtering false interactions, and imputing missing distal and proximal interactions. The latent representations learned by DeepLinc are also used for inferring the signature genes contributing to the cell interaction landscapes, and for reclustering the cells based on the spatially coded cell heterogeneity in complex tissues at single-cell resolution.

**Supplementary Information:**

The online version contains supplementary material available at 10.1186/s13059-022-02692-0.

## Background

The physiological functions of multicellular tissues are not only defined by heterogeneous cells forming these tissues but are also highly dependent on complicated local and distal cell-cell interactions [1, 2]. Furthermore, it is increasingly recognized that many of the intracellular activities of each single cell are closely related to its interactions with the multicellular context [3]. The intrinsic gene expression profile of each single cell is both a consequence and a defining factor of the complicated cell interaction network in physiological contexts [4, 5].

Recent advances in various spatially resolved transcriptome profiling techniques have made it possible to measure gene expression profiles at single-cell or subcellular resolution while simultaneously retaining information on the spatial locations of cells. These techniques include in situ sequencing methods, such as FISSEQ [6] and STARmap [7]; imaging methods based on fluorescence in situ hybridization (FISH), such as MERFISH [8], seqFISH [9], SPOTs [10], and osmFISH [11]; spatial barcoding techniques, such as Slide-seq [12], HDST [13], and DBiT-seq [14]; and laser capture microdissection (LCM) combined with flow cytometry methods, such as GEO-seq [15] and TSCS [16]. Due to different experimental designs, the coverage of these transcriptome profiles ranges from tens to thousands of genes.

Spatially resolved transcriptome profiles provide insightful resources for understanding the cellular organization patterns in multiple types of tissues and organs, such as the central nervous system [13, 17, 18], developing human heart [19], and tumors [13, 20]. However, these snapshots of tissue sections are still incomplete observations of the multicellular organization and the potential local interactions between geometrically adjacent cells. It remains a major challenge to infer full cell interaction networks, including distal cell-cell interactions, which are potentially mediated by a broad range of different mechanisms. Various methods are currently available for spatially resolving predefined cell types with bulk RNA-seq or scRNA-seq data and for inferring cell-cell interactions based on known ligand-receptor pairs or other predefined features and references [21–25]. However, methods for the de novo reconstruction of cell interaction networks in an unbiased and more comprehensive manner by taking full advantage of spatial transcriptome profiles at single-cell resolution are still lacking.

In addition, due to various technical limitations, spatially resolved single-cell transcriptome profiles suffer to different extents from data imperfections such as too many missing values, batch effects, biased and low coverage, and high noise levels [26, 27], which necessitate methods with a sophisticated design for mining cell-cell interactions from such imperfect and incomplete snapshots. Ideally, such methods should be capable of reducing the noise of spatial transcriptome data, reconstructing existing interactions, restoring missing interactions (including distal interconnections), and mining latent features related to cell-cell interaction landscapes.

The nonlinear, high-dimensional, sparse and multimodal features of single-cell spatial transcriptome data make it a proper and feasible target of the deep learning strategy. Multiple deep learning models have been applied for various tasks with single-cell spatial transcriptome data, for example, integration of histology to define spatial domains and predict local gene expressions with convolutional networks [28, 29], imputation of spatial transcriptome profiles by graph-regularized tensor completion [30], inference of gene-gene interactions with convolutional networks [31], and integration of sc/snRNA-seq data with nonconvex optimization to resolve in situ cell clusters [32]. These methods serve as powerful frameworks for learning from multimodal data and resolving the spatial cell and gene organizations in tissues [33]. However, none of them were designed to directly uncover the cell-cell interactions that shape the tissue organization and define tissue physiological functions.

Deep generative models such as generative adversarial networks (GANs) and variational autoencoders (VAEs) have been proven to be powerful tools for leveraging latent features and modeling high-dimensional scRNA-seq data for various tasks, such as denoising [34], clustering [35], dimension reduction [36], and missing value imputation [37]. In the present study, we adapted another type of deep generative model, variational graph autoencoders (VGAEs) [38], for encoding cell-cell interaction features from spatial single-cell transcriptome data and eventually regenerating full cell-cell interaction landscapes. VGAE is a recently developed state-of-the-art machine learning algorithm in which the core of variational autoencoders has been adapted [39] and extended for graph representation learning [40]. Our method, referred to as DeepLinc (deep learning framework for *l*andscapes of *in*teracting *c*ells), uses the VGAE model to integrate and learn from the two dimensions of information (i.e., cell interactions and gene expression profiles) during the encoding phase. Furthermore, the adversarial strategy was applied to force the encoder to explicitly approximate the Gaussian distribution [41]. The decoding phase then uses the latent representation learned during encoding to reconstruct a cell-cell interaction graph.

Specifically, DeepLinc assumes that the neighboring cells should be much more likely to have some types of interactions than randomly picked non-neighboring cells that are far away from each other. Therefore, for a particular tissue region, the neighboring cell pairs comprise an incomplete and potentially noisy observation of a subset of the full cell-cell interaction network. The main task of DeepLinc is to learn from this subset of cell-cell interactions, extract the underlying features of single-cell transcriptome profiles, and finally, regenerate a more unbiased and complete landscape of cell-cell interactions, which would include both proximal and distal interactions. To the best of our knowledge, DeepLinc is the first framework of its kind to apply a deep generative model for recovering cell interactions from spatial single-cell transcriptomic data.

Based on a wide array of tests with real and simulated data, DeepLinc demonstrated its high efficiency in learning from imperfect and incomplete spatial transcriptome data, filtering false interactions, and inferring missing distal and proximal interactions. The reconstructed full networks of cell interactions exhibited high physiological relevance. Such de novo reconstructions of cell interaction networks do not depend on prior knowledge of cell types, ligand-receptor pairs, or cell interaction mechanisms. Furthermore, the interrogation of the pipeline revealed signature genes that are potentially involved in shaping cell interaction landscapes. Finally, the reconstructed cell interaction landscapes, which are presumably more complete than the original snapshots of cell spatial organization, categorized hub cells and partitioned cells into further subclusters based on both the features of cell interaction landscapes and transcriptome profiles. These new insights could greatly aid in elucidating the biological relevance and potential machinery underlying cell organization patterns in physiological contexts. In summary, as a specially designed method based on deep learning, DeepLinc is of unique value for mining the latent features of cell interaction landscapes and, thus, for taking full advantage of previous and future spatial transcriptome profiling data at single-cell resolution.

## Results

### The DeepLinc model and the datasets used for testing

**Fig. 1:**
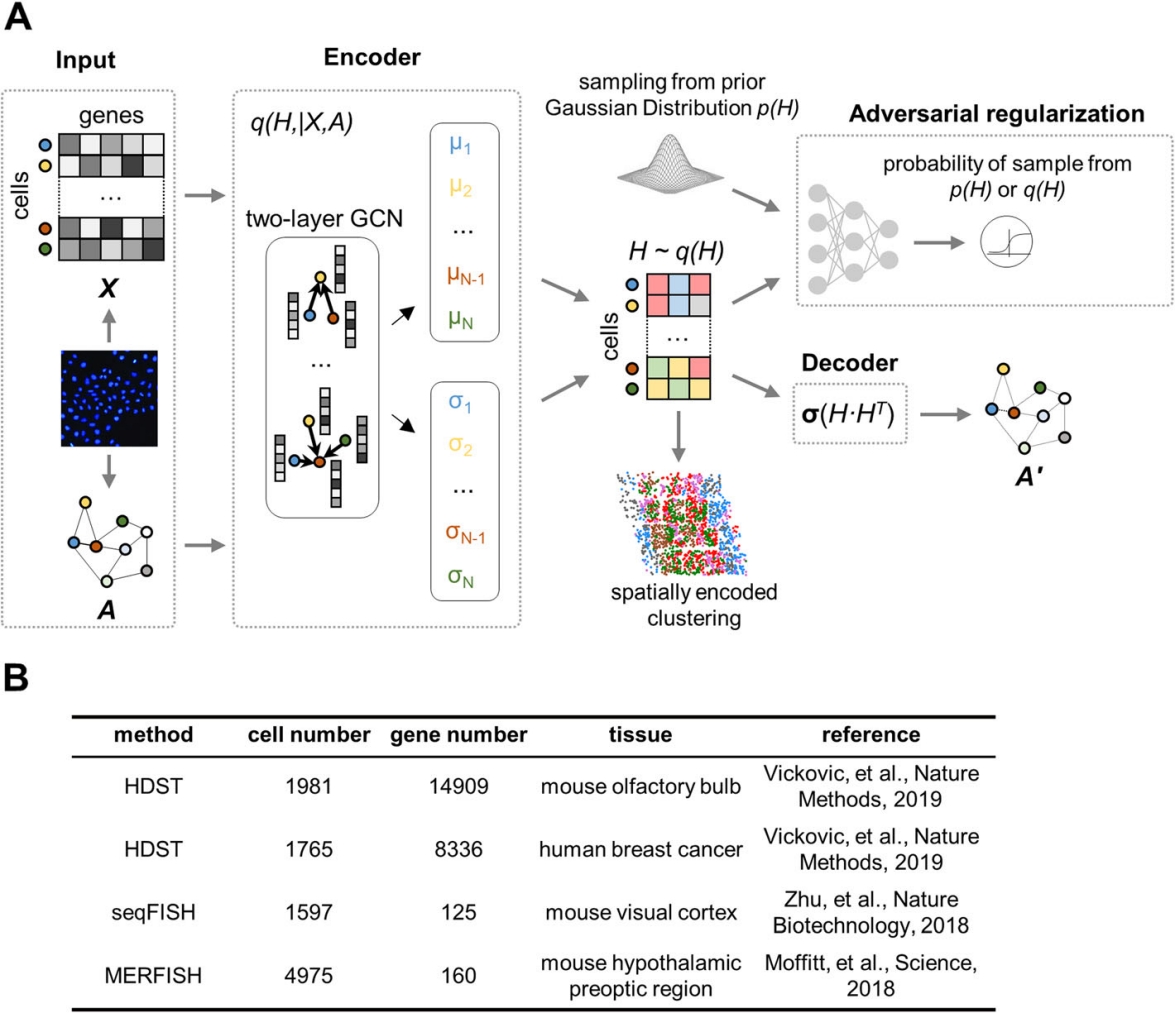
Overview of DeepLinc and the datasets. **A** Schematic description of the DeepLinc pipeline. DeepLinc consists of an encoder, a decoder, and an adversarial regularization module in the VGAE framework. The encoder is a two-layer graph convolutional network (GCN) and the decoder is a Sigmoid function for the dot product of latent variables. The single-cell transcriptome profile and the cell adjacency matrix derived from a single-cell spatial transcriptome dataset serve as inputs of DeepLinc. The output of DeepLinc is a new matrix (A’) presenting the reconstructed cell-cell interaction network. In addition, DeepLinc also uses the latent information of cell interaction landscapes and gene expression profiles for the visualization and clustering of single cells. **B** Statistics of the 4 datasets used for reconstruction of cell interaction networks by DeepLinc

We assume that in a solid tissue the neighboring cells with direct contacts should be much more likely to have some types of interactions than randomly picked non-neighboring cells that are far away from each other. It is well recognized that single-cell transcriptome profiles represent both the driving force and consequences of cell-cell interaction landscapes [4, 5]. DeepLinc combines the VGAE and an adversarial network to learn from single-cell spatial transcriptome profiles and generate a latent distribution capturing the intrinsic associations between cell-cell interactions and the gene expression patterns of single cells (Fig. 1A).

Specifically, adjacent cell pairs defined simply by the geometric closeness between single cells were summarized as an undirected adjacency network in which the nodes represent the cells and the edges indicate the neighboring cell pairs. This network is represented by cell adjacency matrix *A*. The single-cell gene expression profiles, as features of the nodes in *A*, are presented as the matrix *X* (Fig. 1A). The graph-structured data (*A*) and the node features (*X*) are fed into the VGAE consisting of two graph convolutional layers. As the output of this variational graph convolutional network (VGCN) encoder, the latent representation (*H*) captures the characteristics of a single cell itself and its neighboring cells. In addition, *H* is further constrained by an adversarial regularization module from a prior Gaussian distribution (Fig. 1A). Next, with the information learned above, the decoder performs a dot product operation on *H* to generate a new adjacency matrix (*A’*) presenting the reconstructed cell-cell interaction network (Fig. 1A). On the other hand, the vectors of *H*, which represent the latent information of cell interaction landscapes and gene expression profiles, could be extracted for the visualization and clustering of single cells.

DeepLinc was applied to 4 published spatial transcriptomic datasets obtained from the mouse visual cortex [17], the preoptic region of the mouse hypothalamus [18], the mouse olfactory bulb [13], and human breast cancer [13] (Fig. 1B). Note that the spatial transcriptome profiling techniques and the sparseness of the gene expression profiles vary greatly across these studies (Fig. 1B). In brief, the seqFISH and MERFISH datasets show much lower gene coverage (fewer than 200 genes) than the two HDST datasets (approximately 10,000 genes). However, the latter 2 high-throughput datasets are very sparse, and large numbers of genes were not detected in all the single cells in these datasets.

### Modeling of cell interactions by DeepLinc and the effects of denoising

Single-cell spatial transcriptome profiles provide snapshots of cell-cell interaction landscapes, which are presumably just small subsets of interactions from complete cell-cell interaction networks and usually contaminated by high levels of noise. Therefore, the ability of DeepLinc to efficiently learn from the provided information on cell spatial organization and single-cell transcriptome profiles, filter out noise, correctly infer latent interactions, and eventually recover complete cell interaction landscapes is a critical benchmark.

DeepLinc was applied to the 4 seqFISH, MERFISH, HDST olfactory bulb, and HDST breast cancer datasets (Fig. 1B). First, cell adjacency maps were defined by assembling the neighboring cells in the tissue sections. Specifically, the distances between each pair of cells (Additional file 1: Fig. S1A) or between each cell and its 3 closest neighbors (Additional file 1: Fig. S1B) were used to assess the overall distance distributions of adjacent cell pairs with potential direct interactions. The distance threshold for defining direct contacts in each tissue section was then determined based on the above distributions (Additional file 1: Fig. S1B). For each cell, only the 3 closest neighbors falling under the distance threshold were defined as direct contacts. The union of the direct contacts of all the cells then constituted the full adjacency matrix. DeepLinc uses neighboring cells with direct contacts as the positive set for learning the transcriptome features related to cell-cell interactions. From a general biological point of view, we think it is reasonable to assume that in a solid tissue, most of the cells in 2-D could directly contact with 3 or more other cells.

**Fig. 2:**
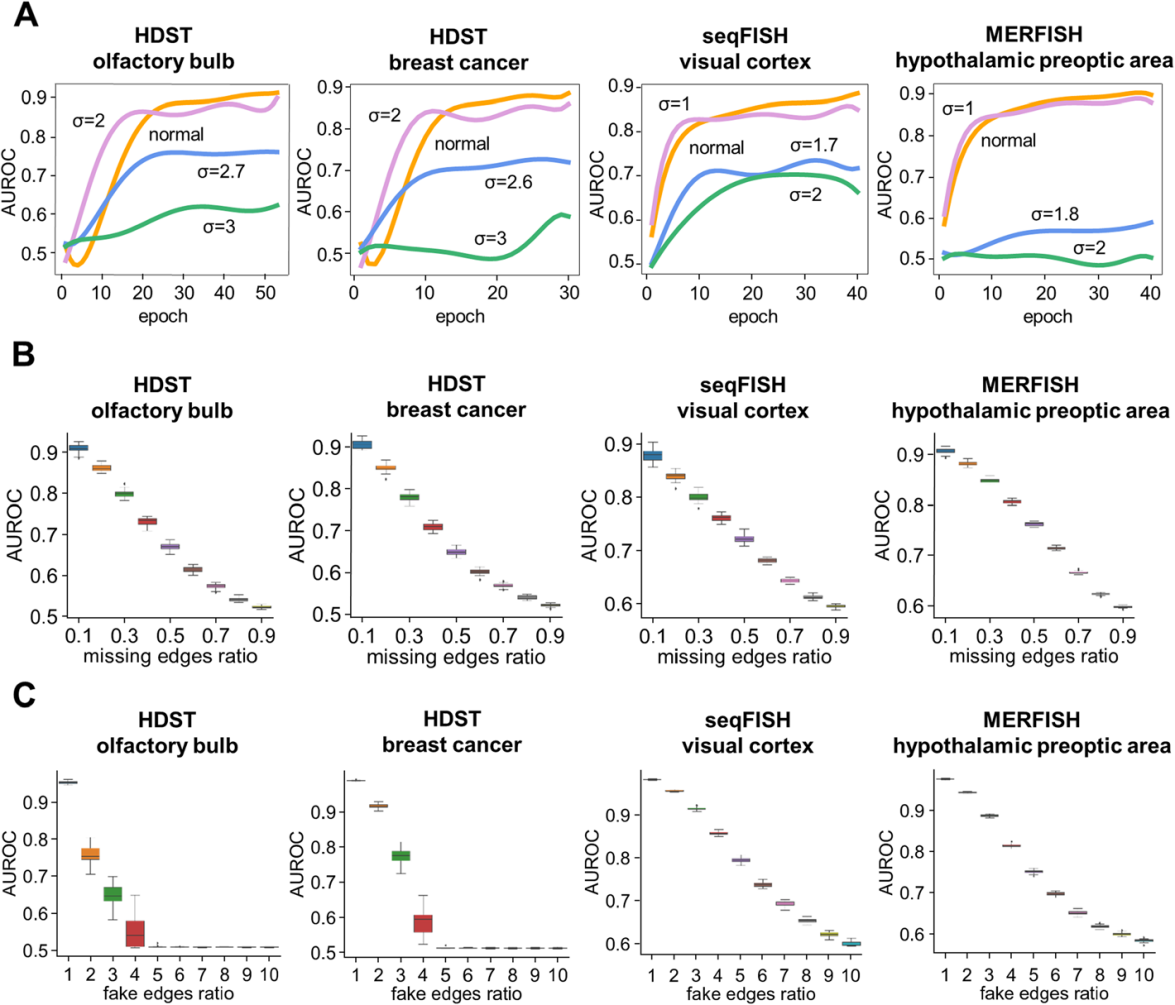
Performance of DeepLinc in reconstructing cell interaction networks. **A** The levels of AUROC at different training epochs of DeepLinc. The original data or data with different levels of random noise in the gene expression profiles were used. Larger *σ* values would result in higher levels of random noise in general (see “Methods” for details). **B** The AUROCs between the randomly removed real edges and originally nonexistent edges. Different proportions of the edges in the original cell adjacency maps were randomly picked and discarded. Then, the remaining edges were used for training the DeepLinc model. This process was repeated 30 times for each proportion of the missing edges to draw a boxplot. **C** The AUROCs between mixed-in fake edges and pre-existing real edges. Different numbers (1- to 10-fold of the size of the original cell adjacency map) of randomly generated fake edges were added into the original cell adjacency map, which was then fed to DeepLinc. This process was repeated 30 times for each level of fake edges to draw a boxplot

The edges of direct contacts were randomly divided into two groups for training (90%) and testing (10%). The negative set for testing, which was 100 times larger than the positive set, was composed of fake edges between two randomly picked non-neighboring cells on the tissue section. For all of the 4 datasets, DeepLinc showed high sensitivity and accuracy in recovering the originally annotated cell-cell interactions, with area under the receiver operating characteristic (AUROC) ranging from 0.8 to larger than 0.9 (Fig. 2A) and false positive rate (FPR) below 5% (Additional file 1: Fig. S2). Notably, DeepLinc modeled the cell interaction networks almost equally well with either the highly sparse high-throughput datasets (HDST olfactory bulb and HDST breast cancer) or the low-throughput datasets (seqFISH and MERFISH).

Next, we tested the tolerance of DeepLinc to artificial noise in the single-cell gene expression data. Specifically, different levels of random noise with Gaussian distributions were introduced on top of the original gene expression profiles of all the cells (Methods). As shown in Fig. 2A, performances of DeepLinc were not compromised by fairly high levels of noise, indicating the robustness of DeepLinc to noise in gene expression data, which is critical when dealing with highly noisy single-cell spatial transcriptome data. However, further corruption of the data dramatically impaired the performance of DeepLinc, illustrating the minimal gene expression profile information needed for DeepLinc to correctly rebuild the cell interaction landscapes.

Single-cell spatial transcriptome data is largely suffering from high sparsity due to large numbers of dropouts, especially for the high-throughput data such as HDST. Therefore, we also generated two more noise models to simulate different types of dropouts (Additional file 1: Fig. S3). In the first model, different percentages of genes were randomly removed from the expression dataset to mimic dropouts of genes, and in the second model, different percentages of the non-zero values were randomly picked and forced to be zero, which mimics dropouts of individual datapoints in single cells. DeepLinc showed high tolerance to the noise from both types of dropouts (Additional file 1: Fig. S3). In general, the performances of DeepLinc were still fairly good with the dropout ratio of 50%. As well expected, more severe dropouts would strongly reduce the accuracy of DeepLinc. In summary, these tests confirm the advantage of DeepLinc in extracting the latent information of cell-cell interactions from the highly sparse and noisy spatial transcriptome profiles.

DeepLinc also demonstrated relatively stable and high levels of fitting (AUC 85~95%) with different sizes of tissue sections that were randomly picked from the original frame of the tissue sections and therefore included fewer cells than the original data (Additional file 1: Fig. S4). The performance of DeepLinc was still fairly good with small tissue sections containing as few as 500 cells, indicating the efficient learning capability of DeepLinc. This is a useful feature for dissecting the cell organization of ultrafine tissue sections.

We also tested DeepLinc for its capability to recover cell-cell interaction landscapes with different proportions of missing interactions. Specifically, certain percentages of the existing edges were randomly removed from the original cell-cell interaction network. The remaining network and the single-cell transcriptome data were then used for training the DeepLinc model. Ultimately, AUROC values were calculated to evaluate the performance of DeepLinc in correctly distinguishing the arbitrarily removed interactions (positives) and the originally nonexistent interactions (negatives). As shown in Fig. 2B, DeepLinc exhibited high precision in imputing the missing positive interactions. For example, even when half of the interactions were lost in the input, DeepLinc still managed to recover these missing edges with 65–75% precision (Fig. 2B). This indicates that DeepLinc can indeed efficiently learn from limited and incomplete networks of cell interactions to reinstate missing edges. Such an imputation and prediction capability is critical for applications of DeepLinc for the inference of full cell interaction landscapes from the incomplete snapshots generated from spatially resolved single-cell transcriptome profiles.

In addition to the missing interactions (i.e., false negatives), false annotations of cell-cell interactions (i.e., false positives) that are fed to DeepLinc are another major issue to address. To test the robustness of DeepLinc in correctly recovering cell interactions with random false interactions (i.e., the denoising capability), we added fake edges between randomly picked cell pairs into the original cell interaction network. Taking such highly noisy data as the input, DeepLinc can still nicely distinguish the originally nonexistent edges and the real pre-existing edges (Fig. 2C). Even when fake edges were artificially added in numbers as high as several-fold the original number of cell interactions, DeepLinc still managed to filter out the extensive noise and recover the predefined cell interactions with fairly good precision (Fig. 2C). This indicates the high tolerance of DeepLinc to random false interactions and its efficient denoising effects

### Reconstructed cell-cell interaction landscapes

**Fig. 3:**
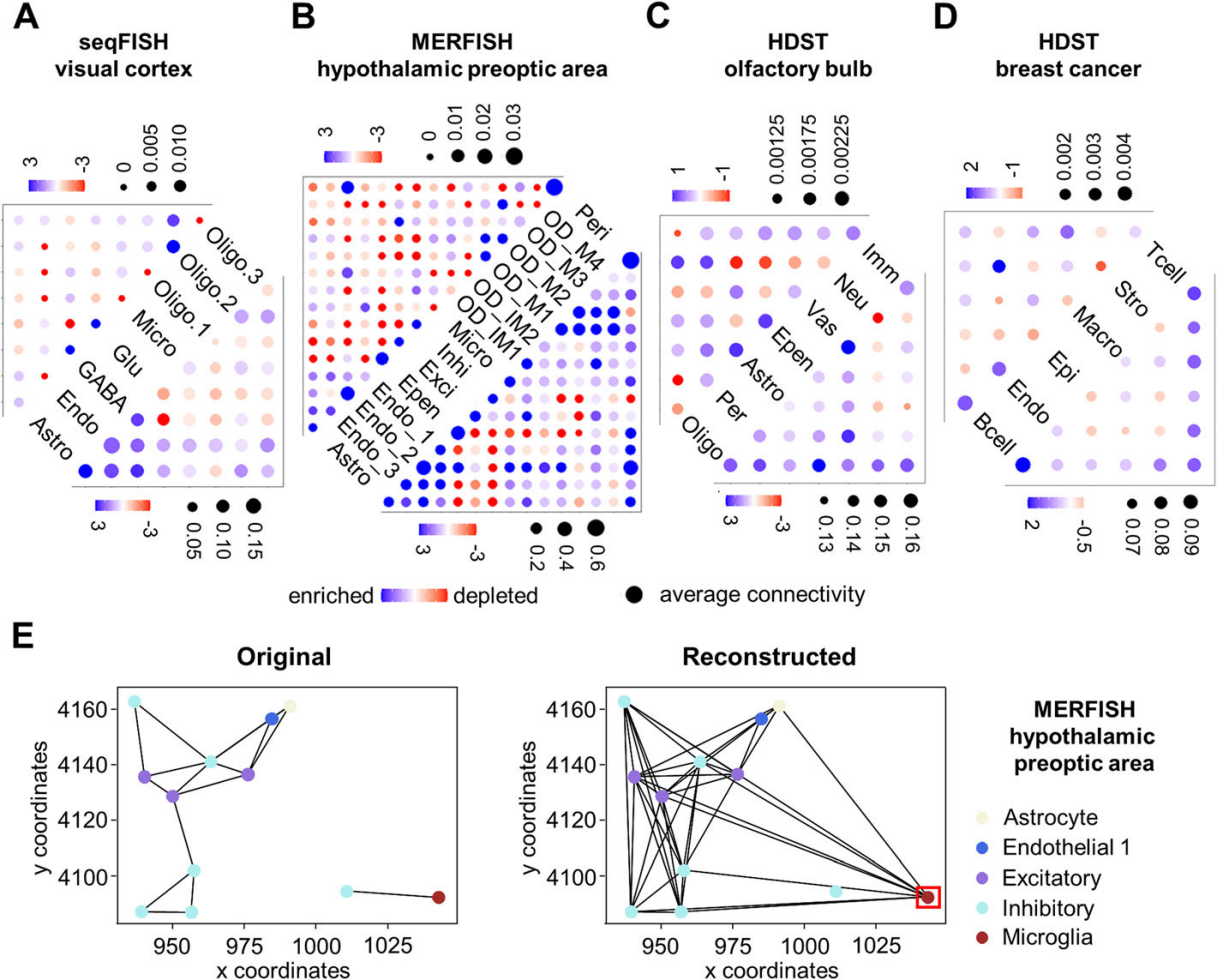
Landscapes of cell interactions reconstructed by DeepLinc. **A–D** Evaluation of the over- or under-representation of particular types of interactions in the original cell adjacency networks (left) and in the reconstructed cell interaction networks (right) with the 4 datasets. The results are represented as dot heatmaps showing the interaction relationships between cell types. Dot size represents the connectivity between two cell types, calculated by the number of connections divided by the product of the cell numbers. The color of the dots represents significance (−log_10_P) determined by comparing with the permutation test, in which positive values indicate enrichment and negative values indicate depletion. **E** Original and reconstructed cell interaction networks in a representative tissue section from MERFISH dataset of hypothalamic preoptic area. The red box indicates a microglia cell with distal interactions with many other cells in the reconstructed networks

The landscapes of the reconstructed and the original cell-cell interaction networks were interrogated with a permutation test as described previously [21, 24, 42] to evaluate the enrichment or depletion of certain types of interactions in a network by comparison with randomly assembled networks with similar scales. As shown in Fig. 3A–D, the cell-cell interaction landscapes reconstructed by DeepLinc exhibited substantial differences from the original cell adjacency networks composed of proximal cell pairs with direct contacts.

For the visual cortex data of seqFISH, relative to the original network, the reconstructed network reinforced the interactions between the astrocytes and the two types of neurons (glutamatergic and GABAergic) (Fig. 3A). Interactions between epithelial cells and almost all the other cell types were significantly enriched in the reconstructed network. On the other hand, despite the high count, the interactions among the glutamatergic neurons appeared depleted when compared to random networks (Fig. 3A), whereas such interactions were highly enriched in the original cell adjacency network. Therefore, the reconstructed network indicates high selectivity of the interactions among the excitatory glutamatergic neurons.

For the MERFISH data of the mouse hypothalamic preoptic region, the reconstructed network strengthened the interactions within endothelial cells and between endothelial cells and astrocytes (Fig. 3B), similar to the observations based on the mouse visual cortex data discussed above. However, unlike the visual cortex, the mouse hypothalamic preoptic region showed enriched interactions between excitatory (mainly glutamatergic) and inhibitory (GABAergic) neurons in the reconstructed network. In addition, the interactions within excitatory neurons were strongly enriched (Fig. 3B), whereas the opposite observation was obtained from the visual cortex data (Fig. 3A). Therefore, the above results revealed both common and tissue-specific cell interaction patterns in the two types of CNS systems. Finally, unlike the original cell adjacency network, the reconstructed network of the mouse hypothalamic preoptic region showed strong enrichment according to the interactions among the mature oligodendrocytes, the interactions between microglia and many other cells, and the interactions between pericytes and other cells (Fig. 3B).

The cell interaction network reconstructed from HDST data of the olfactory bulb showed more enriched interactions between oligodendrocytes and most of the other cell types, whereas the interactions between immune cells and other cells were reduced relative to the random networks (Fig. 3C). In addition, the interactions among vascular cells and glial cells (peripheral glia, astrocytes, and ependymal cells) were specifically intensified in the reconstructed network (Fig. 3C), indicating strong internal interactions within the complex blood–brain barrier composed of these cells [43–45].

Finally, for the breast cancer tissue profile data obtained with HDST, the reconstructed network exhibited strong interactions between lymphocytes (T cells and B cells) and almost all the other cell types (Fig. 3D). In contrast, the interactions between stromal cells and endothelial cells were enriched in the original network but depleted in the reconstructed network, suggesting high specificity of such interactions (Fig. 3D).

In summary, by learning from the cell adjacency maps defined simply by the spatial organization of cells, which are incomplete, sparse, and noisy, DeepLinc performs the imputation of missing interactions and the filtration of potentially false connections. Ultimately, the reconstructed interaction networks are significantly enlarged and reshaped, which should recapitulate the cell interaction landscapes in a more comprehensive and precise manner.

As shown by the local regions from different tissues in Fig. 3E and Additional file 1: Fig. S5 as examples, small numbers of the originally defined direct cell interactions were removed, and new interactions were added by DeepLinc. The reconstructed networks had more hierarchical and modularized structures. Interestingly, in the two examples of visual cortex and hypothalamic preoptic area, microglia showed dense connections with many other cells, including neurons, astrocytes, endothelial cells, and oligodendrocytes, thereby serving as hub cells (Fig. 3E, Additional file 1: Fig. S5C), which nicely recapitulated the wide-ranging functions of microglia and their well-acknowledged interactions with other cell types in the CNS [46]. Such insight was missing in the original cell-cell interaction network.

### Distal interactions in the reconstructed cell networks

The probability of cell-cell interactions predicted by DeepLinc showed slightly negative correlations with geometric distances in general, and significant numbers of distal cell pairs presented high probabilities of interactions (Additional file 1: Fig. S6). Such distal interactions were missing in the original cell adjacency map, and DeepLinc demonstrated its capability to recover cell-cell interactions that are not restricted by spatial proximity.

**Fig. 4:**
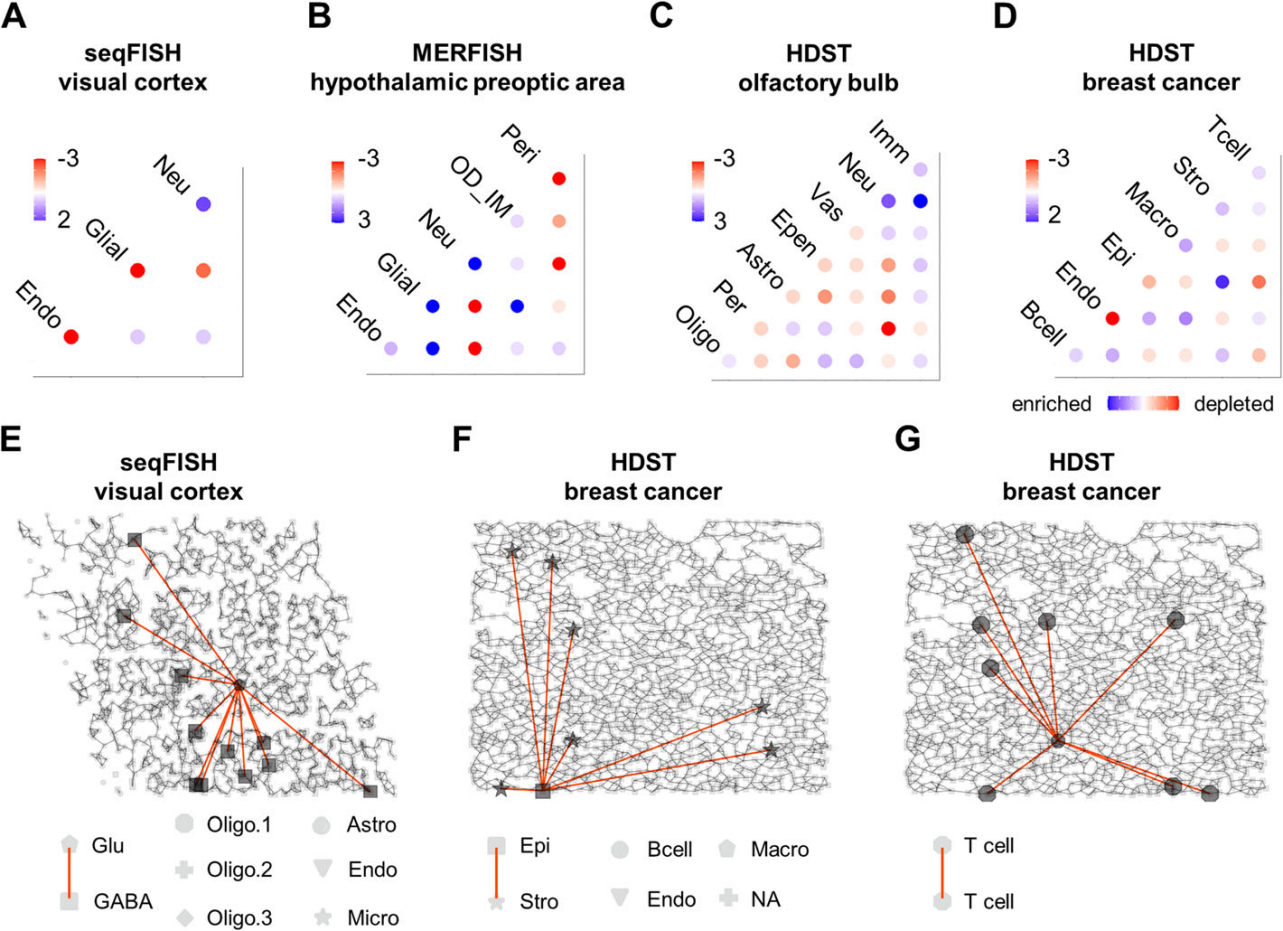
Landscapes of distal interactions in the reconstructed cell networks. **A–D** Evaluation of the over- or under-representation of particular types of distal interactions in the reconstructed cell interaction networks with the 4 datasets. The results are represented as dot heatmaps showing the enrichment or depletion of the distal interactions between certain cell types. The color of the dots represents significance (−log_10_P) determined by comparing with the permutation test, in which positive values indicate enrichment, and negative values indicate depletion. **E–G** Examples of distal interactions between a glutamatergic neuron and GABAergic neurons (**E**), between an epithelial cell and stromal cells (**F**), and between a T cell and other T cells (**G**). The proximal interactions between neighboring cells are shown as backgrounds

The newly recovered distal interactions showed distinct patterns of participating cell types. For example, in the seqFISH study of the mouse visual cortex, distal interactions were highly enriched between neurons (Fig. 4A). An example is provided in Fig. 4E to show the interactions between a glutamatergic neuron and other GABAergic neurons. By contrast, distal interactions were depleted between endothelial cells (Fig. 4A). This is in line with the notion that endothelial cells form a tight one-cell-thick interior interface in blood vessels, and it is not surprising to see a lack of distal interactions between endothelial cells. In addition, a similar depletion of distal interactions between endothelial cells was observed in the HDST breast cancer data (Fig. 4D).

In the hypothalamic preoptic region dataset obtained with MERFISH, the newly reconstructed distal interactions were depleted between pericytes and other cell types (Fig. 4B) relative to the expectation based simply on chance. Indeed, embedded in the basement membrane of blood capillaries, pericytes directly interlock and communicate with endothelial cells [47]. Long-distance interactions between pericytes and other cell types are indeed not expected.

Being similar to the seqFISH study of the mouse visual cortex, an enrichment of the distal interactions between neurons was also observed in the MERFISH data of hypothalamic preoptic region and HDST olfactory bulb dataset (Fig. 4B, C). In addition, in both the hypothalamic preoptic region and the olfactory bulb, the newly reconstructed distal interactions were depleted between neurons and glial cells (Neu-Glial in Fig. 4B, and Neu-Epen/Astro/Per/Oligo in Fig. 4C) relative to the expectation based simply on chance. By contrast, in the olfactory bulb, immune cells showed a broad spectrum of long-range interactions with almost all cell types, especially with neurons, indicating abundant nerve-immune circuits (Fig. 4C).

In the HDST breast cancer data, the newly recovered distal interactions were enriched with interactions between epithelial and stromal cells (Fig. 4D). An example is provided in Fig. 4F to show the interactions between an epithelial cell and multiple stromal cells. This was not surprising given the frequent reports of stromal-epithelial cell interactions mediated by cytokines and extracellular vesicles [48–50], which confer high potential for remote interactions [51, 52]. Similarly, T cells or B cells heavily rely on long-range communication via cytokines, a notion reflected by the distal interactions enriched among T or B cells in the reconstructed network (Fig. 4D, and an example illustrating interactions between T cells is given in Fig. 4G). In contrast, the long-distance interactions were depleted in interactions that are highly dependent on direct cell contacts (for example, antigen presentation), such as the interactions between T and B cells, T cells and epithelial cells, T cells and macrophages, B cells and epithelial cells, and B cells and macrophages (Fig. 4D).

In the HDST breast cancer data, the epithelial cells were mostly tumor cells. Taking 4 randomly picked T cells and epithelial cells as examples, Additional file 1: Fig. S7 shows the distances between the interacting cell pairs, which were generally much shorter than those from the randomly shuffled networks. Therefore, DeepLinc recovered the enriched interactions between T cells and tumor cells (Fig. 3D), which were strongly limited to proximal and not distal interactions (Fig. 4D, Additional file 1: Fig. S7).

Interestingly, in contrast to epithelial cells, endothelial cells were more involved in distal interactions with immune cells (Fig. 4D). Indeed, it has been frequently reported that endothelial cells secrete chemokines to facilitate the transmigration and homing of immune cells into tumor tissues [53, 54].

### DeepLinc identifies signature genes shaping the cell interaction landscapes

Next, we further interrogated the DeepLinc model to infer the signature genes that play important roles in shaping the comprehensive landscapes of cell-cell interactions. In brief, the expression profile of a particular gene was shuffled across all the single cells, and the new transcriptome data and the cell adjacency network were then fed to DeepLinc. The AUPRC calculated from the cell network reconstructed by DeepLinc with these modified data was then compared to the original AUPRC with the unmodified gene expression data. The reduction, defined as the sensitivity score, indicates the sensitivity of the DeepLinc model to the particular gene being shuffled and therefore serves as an assessment of the potential contribution of the gene to the cell-cell interaction landscape. The same procedure was performed for each gene one at a time to obtain the sensitivity scores of all the genes (Additional file 1: Fig. S8).

**Fig. 5:**
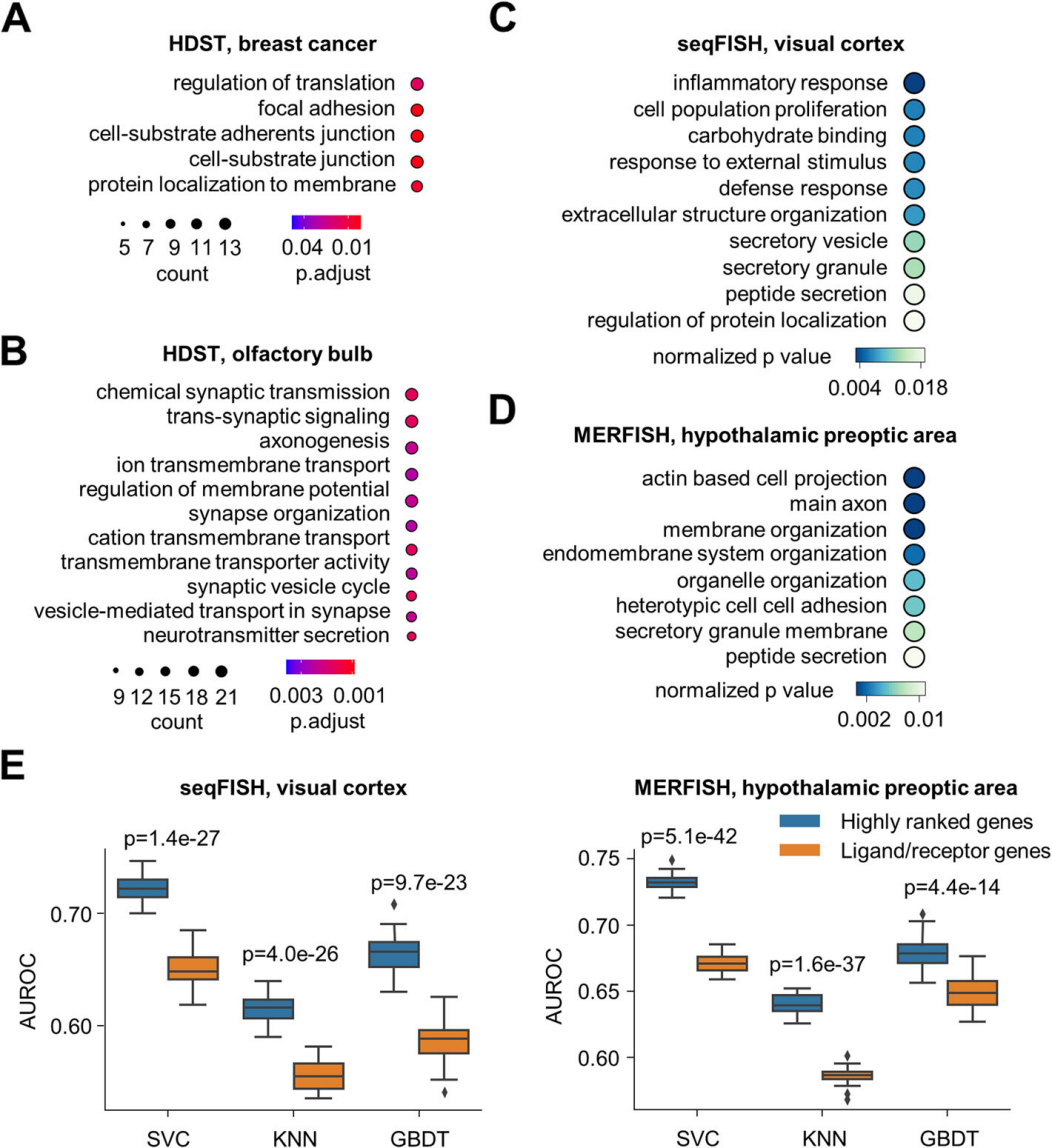
Biological processes enriched by the signature genes in shaping the cell interaction networks. **A**, **B** For the two high-dimensional datasets of HDST, the biological processes involving the top-ranked genes were obtained through Gene Ontology Enrichment Analysis. **C**, **D** For two low-throughput datasets (seqFISH and MERFISH), due to the limitation of gene numbers, all the genes were sorted by their sensitivity scores, and gene set enrichment analysis was performed. **E** Prediction of cell interactions with genes annotated as ligands or receptors and the same number of top-ranked signature genes identified by DeepLinc in the seqFISH (left) and MERFISH (right) datasets. Simple machine learning models, including support vector classification (SVC), K-nearest neighbor (KNN), and gradient boosting decision tree (GBDT) models, were used for this test. The originally defined cell adjacency map was used as a positive set of cell interactions, and randomly selected nonadjacent cell pairs with the same numbers were used as a negative set. This process was repeated 30 times with different training and testing set assignments. ***P***-value were obtained with Student’s ***t*** test

The top-rated genes with the highest sensitivity scores were indeed enriched by multiple molecular and cellular processes related to cell-cell interactions (Fig. 5A–D). For example, 205 genes (Additional file 2: Table S1) showed outstanding sensitivity scores with the HDST breast cancer data, and these genes were involved in the processes of cell-substrate adhesion, junction formation, and protein targeting to the membrane (Fig. 5A, and the full list of functional categories supplied in Additional file 1: Fig. S9A). SPATA2, which showed the highest sensitivity score (Additional file 1: Fig. S8B), mediates the recruitment of CYLD to the LUBAC complex [55, 56], thereby playing a crucial role in the TNFR1- and NOD2-signaling pathways. The rest of the top 10 genes listed in Additional file 1: Fig. S8B include PKHD1 (transmembrane or secreted protein, involved in ciliogenesis, cell-cell and cell-matrix adhesion, and calcium ion homeostasis), S100PBP (binding partner of the calcium sensor S100P, involved in cell adhesion) [57], PSD4 (GEF for ARF6, involved in endocytosis), TRIM5 (capsid-specific restriction factor, involved in innate immune signaling and autophagy), ADAP2 (GTPase-activating protein for ARFs, localizing to the plasma membrane, showing phospholipid binding activity and mediating TCR signaling), and CCL26 (C-C motif chemokine ligand for CCR3).

For the other HDST high-throughput spatial transcriptome dataset of the mouse olfactory bulb, the top-rated genes (Additional file 3: Table S2) were more specifically involved in the cell-cell interactions of the nervous system, such as synaptic transmission, ion transmembrane transport, axonogenesis, synaptic vesicle activities, and transsynaptic signaling (Fig. 5B, and the full list of functional categories supplied in Additional file 1: Fig. S9B).

For the two low-throughput datasets of the mouse visual cortex (seqFISH) and mouse hypothalamic preoptic region (MERFISH), we performed gene set enrichment analysis (GSEA) to identify the processes involving more of the top-rated signature genes for the cell interaction landscapes (Fig. 5C, D, and the full lists in Additional file 1: Fig. S10). In the mouse visual cortex, these processes included the inflammatory response, response to external stimulus, defense response, extracellular structure organization, signal release, secretory vesicle processes, and peptide secretion (Fig. 5C). In the hypothalamic preoptic region, the identified processes included actin-based cell projection, axon, membrane organization, cell-cell adhesion, secretory granule, and peptide secretion (Fig. 5D).

Together, the above results indicate the capability of the DeepLinc model to mine transcriptome signatures that are directly or indirectly associated with cell interaction landscapes. The high-impact genes revealed by the interrogation of the DeepLinc pipeline provide insights into the key processes defining different types of cell interactions in specific tissue contexts. Interestingly, compared to the annotated ligand and receptor genes, equal numbers of the top-rated signature genes were much more efficient in distinguishing interactive and noninteractive cell pairs with simple classifiers in both the mouse visual cortex and mouse hypothalamic preoptic region datasets (Fig. 5E). This once again suggests that the information on cell-cell interactions is largely coded in the transcriptome and not limited to specific ligand and receptor genes, a notion that lays the foundation of DeepLinc for the inference of cell interaction landscapes with spatial transcriptome data.

### DeepLinc identifies multicellular domains informing organizational tissue structures

As a deep data-generative model, DeepLinc generates a latent representation (*H*), which captures both the characteristics of the single-cell transcriptome profile and its position in the cell-cell interaction landscape. Therefore, we used this latent information of the cells learned by DeepLinc to redefine the multicellular spatial domains in heterogeneous tissues.

**Fig. 6:**
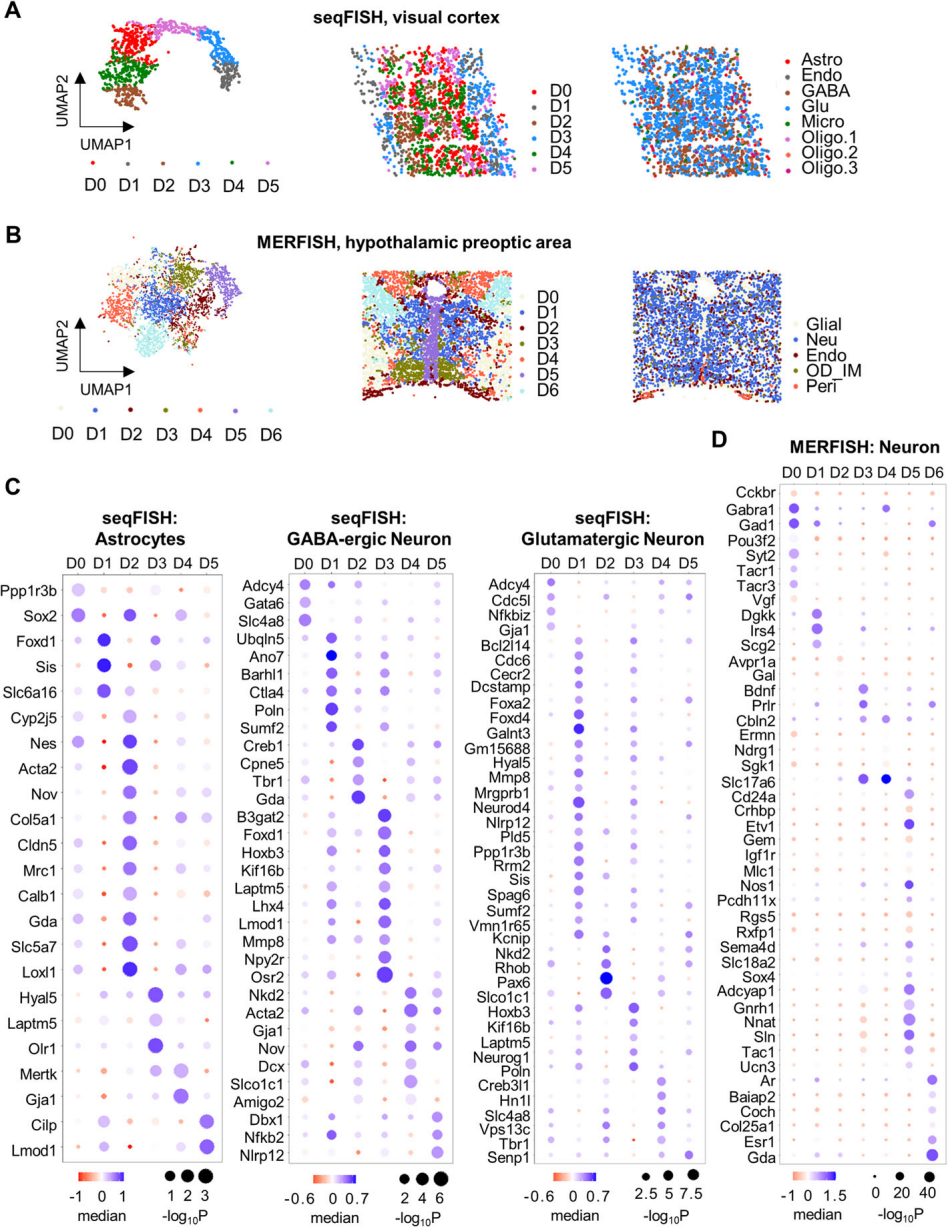
Spatial domains defined by reclustering of cells based on latent vectors recovered by DeepLinc. **A**, **B** The UMAP graph and spatial locations of different spatial domain clusters and cell types. The spatial domain clusters were acquired by the *k*-means method with latent vectors of the single cells. **C**, **D** Spatially specific gene expression signatures of homologous cells reclustered by DeepLinc. The heatmaps show the differential expression of domain-specific genes. The dot size represents the significance of the one-versus-rest test (Mann–Whitney ***U*** test). The color indicates the median value of the normalized ***z***-scores of gene expression in each cluster

For example, in the seqFISH data of the mouse visual cortex, the embedding vectors in *H* for each cell were used for unsupervised K-means clustering, resulting in 6 subgroups of cells (Fig. 6A). The optimal cluster number was defined by Calinski-Harabasz scores [58] (see methods for details). These cell clusters were largely different from the previously annotated cell types based only on known marker genes (Fig. 6A). Instead, the new cell groups resembled the multilayered organization of the heterogeneous cells in the mouse visual cortex [17] (Fig. 6A), which reflected the spatially restricted developmental trajectory from the inner to outer regions of the tissue. Similarly, for the MERFISH dataset of the hypothalamic preoptic area, the same analysis classified the tissue section into 7 subregions (Fig. 6B), which were again highly correlated with the original anatomy.

Next, for the same types of cells that are allocated to these different spatially coded cell clusters, differential expression analysis identified domain-specific signature genes. Taking astrocytes and GABAergic and glutamatergic neurons in the mouse visual cortex as examples, the same types of cells allocated in the 6 domains showed significantly differential expression patterns of some signature genes (Fig. 6C). The details of these patterns were well in line with the inner-outer structure of the mouse visual cortex. However, the same types of cells located in the same layer were also partitioned into different cell clusters (e.g., Clusters D1 and D3) (Fig. 6A). Although the astrocytes, GABAergic neurons, and glutamatergic neurons in D1 and D3 showed similar patterns of gene expression in general, some genes were differentially expressed, indicating the differences between D1 and D3 (Fig. 6C). This again indicates that DeepLinc captures features from both gene expression profiles and spatially coded cell interaction landscapes. Similarly, for the MERFISH dataset of the hypothalamic preoptic area, neurons allocated into different domains also showed differentially expressed signature genes (Fig. 6D).

In summary, DeepLinc rebuilt more comprehensive cell interaction landscapes by learning from spatially resolved single-cell transcriptome profiles. The latent information on spatially dependent cellular features redefined the geometric domains of tissues composed of heterogeneous cells. These results therefore provide unique insights into the spatially coded cellular organization underlying the potential physiological relevance of cell interaction landscapes.

## Discussion

As a major methodological breakthrough, technologies for profiling spatially resolved transcriptomes have provided unprecedented opportunities for the dissection of highly heterogeneous cellular organizations in physiological and pathophysiological contexts. However, it has been a major challenge to comprehensively and precisely infer cell-cell interactions based on spatial transcriptome profiles at single-cell resolution. First, spatial transcriptome profiles are random snapshots of very limited numbers of tissue sections. The cellular organization patterns contain information on cell-cell interactions and presumably represent only small collections sampled from the full landscape. Therefore, methods for correctly inferring the complete landscapes of cell interactions by taking advantage of the observed cell geometric distributions and gene expression profiles are urgently needed. Second, current spatial transcriptome data are limited by a series of technical issues, including biased and low gene coverage, low signal-to-noise ratios, high data sparsity under high-throughput techniques, and/or batch effects, thereby necessitating data-mining methods that are highly robust to these technical limitations.

Powered by the strong data representation capacity of the VGAE model, DeepLinc was specifically designed and optimized to denoise and infer missing interactions by efficiently learning from the incomplete sets of cell organization revealed by different methods of single-cell spatial transcriptome profiling. Further analysis of the features mined by DeepLinc helps identify the signature genes shaping cell interaction landscapes and cells with special features, such as hub cells and cells in specific domains of the interaction landscape.

Various methods have been available for inferring the cell-cell interaction landscapes utilizing bulk RNA-seq, scRNA-seq, or spatially resolved single-cell data. These methods are mostly based on predefined or previously annotated ligands, receptors, and downstream target genes [23, 25, 59–61]. These methods have mostly been applied to scRNA-seq data and are not well suited for single-cell spatial transcriptome data, which show very limited and uneven coverage of the ligand and receptor genes due to high sparsity and noise. This necessitates de novo methods, such as DeepLinc, for reconstruction of cell-cell interaction networks in a more unbiased manner independent of prior knowledge such as ligand-receptor pairs. Instead, by assuming that cell interaction potentials have been coded in single-cell transcriptome profiles, DeepLinc is trained by the observed cell-cell connections and then utilized to infer missing interactions. As shown in Fig. 4 and Additional file 1: Fig. S5, DeepLinc recovered great numbers of distal interactions, and meanwhile, DeepLinc removed some of the predefined proximal interactions between neighboring cells. Therefore, DeepLinc is positioned as a data-mining and processing strategy tailored for single-cell spatial transcriptome profiling data, with the goal of recovering the full landscapes of cell interactions from limited observations of tissue sections.

Specifically, DeepLinc uses VGAE to encode the intrinsic characteristics of spatial transcriptome data and decode cell interactions. The dual modal data (i.e., the graph-like cell adjacency map and the high- or mid-throughput transcriptome profile) are integrated at the same time. Here, the design of VGAE constrains the model to put more weight on the key genes by force-fitting the global cell interaction graph signatures. To the best of our knowledge, DeepLinc is the first deep generative learning model for inferring cell interactions from spatial transcriptomic data. Based on a wide array of tests with real and simulated data, DeepLinc demonstrated high efficiency in learning from imperfect and incomplete spatial transcriptome data, filtering false interactions, and inferring missing distal and proximal interactions, thereby suggesting the reliability and effectiveness of the deep learning strategy.

With the DeepLinc model, our sensitivity test of transcriptome profiles suggested signature genes with potentially high impacts on the cell interaction landscape. Importantly, compared to known ligand and receptor genes, these genes showing high sensitivities performed better in simple classification models of cell interactions. This supports our presumption that cell-cell interactions are coded in single-cell transcriptome profiles, rather than simply being defined by ligand and receptor genes. The identification of these signature genes could facilitate the understanding of the various machineries underlying heterogeneous cell interactions and further suggests the possibility of directly predicting cell interactions simply from single-cell transcriptome profiles. Further refined models would be needed for this purpose.

Due to the lack of more detailed information in spatial transcriptome data, DeepLinc does not provide the directionality or strength of the cell interactions. Potentially different modes of cell interactions also cannot be differentiated by DeepLinc in its current form. Finally, similar to most of the deep learning models based on GAE, it is difficult to efficiently and directly backtrack the important features encoded by the graph convolutional networks used in DeepLinc. Technical advances in spatial transcriptome profiling and breakthroughs in graph convolutional models would help further improve DeepLinc in the future. In addition, if more information becomes available due to future technological advances, DeepLinc could be further expanded to incorporate features such as cell morphology, surface markers, and metabolic profiles. Finally, although this is not the main target of DeepLinc, the framework can be used for inferring single-cell gene expression profiles by using information on cell interactions based on the assumption that the gene expression profiles are partly correlated with local and distal cell interaction patterns [62].

In summary, as a tool for deep data mining, DeepLinc further empowers rapidly emerging spatial transcriptome profiles for the de novo reconstruction of cell interaction maps. This new strategy, facilitated by deep graph convolutional networks, does not rely on any prior knowledge of cells or gene functions. We anticipate that the combination of state-of-the-art spatial transcriptome profiling techniques and an efficient data deep mining framework will greatly facilitate the identification of the biological mechanisms underlying complex cell communication networks with physiological relevance.

## Conclusions

DeepLinc is a novel computational method, with a strategy of deep learning, for de novo reconstruction of cell interaction landscapes from single-cell spatial transcriptome profiles. With simulated and real data, DeepLinc demonstrates high efficiency in learning from imperfect and incomplete spatial transcriptome data, filtering false interactions, and inferring missing distal and proximal interactions. Interrogations of the DeepLinc pipeline reveal signature genes that are potentially involved in shaping cell interaction landscapes. In addition, clustering of cells based on the latent features of cell interactions and transcriptome profiles learned by DeepLinc indicates multicellular domains informing organizational tissue structures.

## Methods

### Datasets of single-cell spatial transcriptome profiles

Four published datasets were used for the current study [13, 17, 18], including mouse visual cortex profiled by seqFISH [17], preoptic region of the mouse hypothalamus by MERFISH [18], mouse olfactory bulb, and human breast cancer by HDST [13] (Fig. 1B). Fewer than 200 genes were profiled by seqFISH and MERFISH, whereas HDST generates high-throughput transcriptome profiles coving around 10,000 genes (Fig. 1B).

The seqFISH dataset was downloaded from the data portal at https://bitbucket.org/qzhu/smfish-hmrf/src/master/. The data was processed according to the procedure described in the original study [17]. The final dataset covers 125 genes measured in 1597 cells.

The MERFISH dataset of the mouse hypothalamic preoptic region was downloaded from https://datadryad.org/stash/dataset/10.5061/dryad.8t8s248^18^. We used the slice region at Bregma+0.11 mm of the animal No. 18, as it contains the largest number of single cells. After removing the ambiguous cells, the final dataset contains 4975 cells, covering 160 genes, five of which were blank controls.

The datasets of mouse olfactory bulb and human breast cancer profiled by HDST were downloaded from the data portal at https://portals.broadinstitute.org/single_cell/study/SCP420 [13]. The olfactory bulb dataset contains 3 sections (CN13_D2, CN24_D1, and CN24_E1) from two mice. We selected a field of CN13_D2 (x:8447~11447, y:5447.5~6447.5), which consists of 1981 nuclei. In total, 14,909 genes were measured among these cells, although a majority of them were not detected in each cell. The breast cancer dataset also includes 3 tissue sections (CN21_E2, CN21_C1, and CN21_D1), which were obtained from a histological grade 3 HER2+ patient. We selected a field of CN21_E2 (x:8464~9664, y:5000~6500) consisting of 1765 nuclei. This data, being very sparse as well, covers 8336 genes.

### Overview of the DeepLinc pipeline

#### The algorithm of DeepLinc

DeepLinc assumes that the neighboring cells in a solid tissue should be much more likely to have some types of interactions than randomly picked non-neighboring cells that are far away from each other. Therefore, for a particular tissue region, the neighboring cell pairs comprise an incomplete and potentially noisy observation of cell-cell interactions sampled from the comprehensive cell interaction landscape. In other words, neighboring cell pairs in tissue sections reveal some cell-cell interactions, whereas large numbers of other interactions are not explicitly shown. We then assume that the features related to cell interactions are encoded in the gene expression profile of each singe cell.

DeepLinc uses neighboring cells with direct contacts as the positive set for learning the transcriptome features related to cell-cell interactions. From a general biological point of view, it is reasonable to assume that in a solid tissue, most of the cells in 2-D could directly contact with 3 or more other cells. From a technical point of view, for the strategies of machine learning, it is critical to minimize the potential false positives in the predefined positive sets for training. Therefore, for each cell, we only used the 3 nearest neighbors to define direct contacts, which we believe is a balanced choice generating enough number of direct contacts for training the DeepLinc pipeline, and at the same time, ensuring few false positives to contaminate the positive training set.

DeepLinc leverages a variational graph autoencoder (VGAE) with an adversarial network for regularization, to infer the unobserved interactions between cells. The entire architecture includes three parts: a variational graph convolutional network (VGCN) encoder, an inner product decoder, and an adversarial module.

Given a cell adjacency matrix *A* and a gene expression matrix *X*, the VGAE learns the embedded features *H* of the cell graph. A two-layer graph convolutional network (GCN) (250-125) is used as the encoder module.

$$ q\left(H|X,A\right)={\prod}_{i=1}^nq\left({h}_i|X,A\right) $$

$$ q\left({h}_i|X,A\right)=N\left({h}_i|{\mu}_i,\mathit{\operatorname{diag}}\left({\sigma}^2\right)\right) $$

Similar to variational autoencoder, *μ* = *GCN*_*μ*_(*X*, *A*) is the matrix of mean vectors and *logσ* = *GCN*_*σ*_(*X*, *A*). As a typical structure, the GCN is defined as $GCN\left(X,A\right)=\overset{\sim }{A} ReLU\left(\overset{\sim }{A}X{W}_0\right){W}_1$ with weight *W*_*i*_ and normalized adjacency matrix $\overset{\sim }{A}={D}^{-\frac{1}{2}}\left(A+I\right){D}^{-\frac{1}{2}}$ to realize layer-to-layer information transmission. We use ReLU as the activation function of the first layer instead of the second layer. The purpose of this step is to learn meaningful embedded features supporting the interaction formation by aggregating information of each cell itself and its interacting neighbor cells.

The inner product decoder *p*(*A*| *H*) = *σ*(*H* · *H*^*T*^) is employed to reconstruct a new cell interaction matrix, which reports probability scores of the interactions between each pair of single cells.

VGAE has exhibited outstanding performance in learning the embedded features of graph elements [38]. However, for single-cell spatial transcriptome data, a major challenge is its high sparsity and data noise. For such non-ideal situations, the standard variational encoder-decoder structure adopted by VGAE does not make more constraints on latent variables, often resulting in underfitting of the sparse data [63]. Here we implement strong regularization on the latent distribution with the adversarial network.

Specifically, the adversarial module is built on a multilayer perceptron (MLP). The architecture is similar to the discriminator of generative adversarial networks (GAN). The adversarial network has two fully-connected hidden layers with ReLU activation (125–150). The output layer uses sigmoid function to judge whether a latent sample *h*_*i*_ is from a prior Gaussian distribution or from VGAE.

Eventually, the DeepLinc pipeline was optimized to maximize the log-likelihood of cell-cell interaction network. The objective of VGAE is to minimize its cost function by optimizing the evidence lower bound (ELBO):

$$ {\zeta}_{ELBO}={E}_{q\left(H|X,A\right)}\left[\mathit{\log}p\left(A|H\right)\right]-{D}_{KL}\left(q\left(H|X,A\right)\left\Vert p(H)\right.\right) $$

For the adversarial module, the loss function is binary cross-entropy.

#### Implementation of DeepLinc

DeepLinc is implemented as a Python package, which is available at https://github.com/xryanglab/DeepLinc. Two files are needed as inputs of the pipeline, one for the cells-by-genes matrix of gene expression data and the other for the predefined cell adjacency matrix. If an enrichment analysis of the interactions between cell populations, an annotation file defining the cell types would be needed.

DeepLinc allows users to specify the node numbers. For datasets with more cells, larger numbers of nodes are recommended to capture the complexity of data better. Although the number of hidden layers for the encoder is also adjustable, extra caution has to be taken, since deeper graph convolutional networks are prone to over-smoothing [64]. In general, the two-layer graph convolutional network has been widely used and proven suitable for most of the tasks. The Adam optimizer [65] with 4e-4 learning rate is used for optimization. The dropout method is also used to ease the overfitting effect.

A new cell interaction matrix is the main output of DeepLinc. The latent representation output by DeepLinc can also be used for unsupervised clustering of the single cells. Implementation of DeepLinc requires the following packages: Numpy v1.16.4, Scipy v1.2.1, Pandas v0.20.3, Matplotlib v3.0.2, Seaborn v0.8.1, Networkx v2.1, Scikit-learn v0.21.2, Tensorflow v1.4.0, Python 3.5.2 (Anaconda3-4.2.0). The DeepLinc model was trained on a single GPU of GTX 1050 or Tesla K80.

### Reconstruction of the cell interaction networks

As introduced above, DeepLinc inferred interaction probability scores for all the cell pairs. Next, cell interaction networks were assembled with the cell pairs with high probability scores. Specifically, different thresholds of the probability scores were tested for identifications of cell interactions. The optimal threshold was then determined so that the newly assembled cell interaction network on the testing set has the highest accuracy for recovering the predefined cell adjacency maps.

### Benchmark of DeepLinc for recovering missing interactions

Different proportions of the edges in the original cell adjacency maps were randomly picked and discarded. The remaining edges were used for training the DeepLinc model. The area under the receiver operating characteristic (AUROC) was calculated to assess the accuracy of the reconstructed interaction networks for recovering the discarded edges. Here equal numbers of the originally non-existing edges were used as true negative sets.

### Benchmark of DeepLinc for differentiating real and fake interactions

Different numbers (1 to 10 folds of the size of the original cell adjacency map) of randomly generated fake edges were added into the original cell adjacency map, which was then fed to DeepLinc. AUROC of the reconstructed network was calculated by taking the pre-existing real edges as positives and the mixed-in fake edges as negatives.

### Benchmark of DeepLinc for the tolerance to random noise in gene expression profiles

Random noise was independently generated and implemented to each gene in each single cell. Specifically, for each gene, the new expression level was regenerated by adding random noise as follows:

$$ \frac{Ex{p}_{noise+}\left(i,j\right)}{Ex{p}_{normal}\left(i,j\right)}={2}^{rand}, $$

$$ \mathit{\operatorname{rand}}\sim N\left(0,\sigma \right) $$

Among them, *Exp*_*noise*+_(*i*, *j*) represents the regenerated expression value of gene *j* in sample *i*, and *Exp*_*normal*_(*i*, *j*) represents the original expression level. The fold change used to simulate noise, after log2 transformation, follows the normal distribution *N*(0, *σ*), whereas different *σ* values would result in different levels of random noise in general.

### Evaluating the over- or under-representation of particular types of interactions

A previously described strategy [21, 24, 42] was used to evaluate the enrichment or depletion of the interactions between different cell types or within a cell type. In brief, we randomly shuffled the reconstructed networks for 1000 times. From these 1000 networks, the numbers of cell interactions of a certain type, e.g., interactions between two types of cells, were used to generate a null distribution. The observed interaction number was then compared to this null distribution, generating a *P*-value indicating how often the random values were higher or lower than the observed ones, which corresponds to either enrichment or depletion of the particular type of interactions in the network. Two-tail test was done for such analysis.

Evaluation of the enrichment or depletion of the distal interactions between certain cell types was done with a similar strategy. For each dataset, we first generate a distance distribution with all the edges in the reconstructed cell interaction network, from which a threshold was determined to classify the distal interactions (Additional file 1: Fig. S6). The same number of interactions was established between randomly picked distal cell pairs. Such procedure was repeated for 1000 times, generating a null background of the distal cell interaction landscapes. Next, the observed number of distal interactions was compared to this null distribution, generating a *P*-value indicating how often the random values were higher or lower than the observed one, which corresponds to either enrichment or depletion of the particular type of distal interactions in the network. Two-tail test was done for such analysis.

### Sensitivity analysis for each gene

Sensitivity analysis was performed to evaluate the potential contribution of a gene to shaping the reconstructed cell interaction landscape. In brief, for a particular gene in the transcriptome profile, the expression profile was shuffled among all the single cells. This new data, with the expression of one gene shuffled at a time, was then fed to DeepLinc, which then generates a new cell interaction network. ΔAUPRC was then calculated by comparing the AUPRC values of the two cell interaction networks built by DeepLinc with the original unperturbed gene expression data and the data with the expression of one gene perturbed. For each gene, the process above was repeated for 30 times, and the average ΔAUPRC was used to quantitatively evaluate the sensitivity of the reconstructed cell interaction network to the gene, thereby defined as the sensitivity score.

For the two high-throughput HDST datasets, distributions of the sensitivity scores of all the genes were prepared, and the biological processes involving the top-ranked genes were obtained with the Gene Ontology enrichment analysis [66]. For two low-throughput datasets of seqFISH and MERFISH, all the genes were sorted by their sensitivity scores, and the Gene Set Enrichment Analysis [67] was performed to acquire the biological processes enriched by the top-ranked genes. The biological processes were filtered by gene numbers (min size=3 and max size=500), resulting in 1240 and 1691 gene sets for the GSEA analysis with the seqFISH and MERFISH data, respectively.

### Prediction of cell interactions with simple machine learning models

For the seqFISH and MERFISH datasets, simple machine learning models including Support Vector Classification (SVC), *K*-nearest neighbor (KNN), and Gradient Boosting Decision Tree (GBDT) were used to test the capability of specified genes for classifications of the interacting and non-interacting cell pairs. The sklearn package in Python was used to build these models. The originally defined cell adjacency map was used as positive sets of cell interactions, and randomly selected unadjacent cell pairs, with the same numbers, were used as negatives.

Sixteen genes annotated as ligands or receptors and the same number of top-ranked signature genes identified by DeepLinc were used for the classification models. Their expression vectors of a pair of cells were concatenated and used as the feature vectors. The combined set of positive and negative cell interactions were randomly divided into 4:1, serving as the training and testing sets, respectively. The three classification models were then applied on the data to predict the interacting cell pairs with the testing set. This process was repeated for 30 times with different training and testing set assignments. The AUROC values were calculated based on the predictions.

### Clustering of the single cells based on the latent representation learned by DeepLinc

Dimension of the latent representation H was set as 10 for clustering of the single cells with the *k*-means method. The Calinski-Harabasz score was used to determine the optimal cluster number. The “umap” package in Python was used for dimension reduction and visualization of the latent vectors of the single cells.

### Differential gene expression analysis between single-cell groups

For the cells of the same type but distributed in different spatial domains, differential expression analysis was performed to identify the upregulated genes specifically in each spatial domain. For each domain, the genes that are significantly upregulated in both one-versus-one and one-versus-rest tests (Mann–Whitney *U* test, *P* < 0.01) were deemed as domain-specific high-expression genes.

## Supplementary Information

**Additional file 1: Figures S1-S10**

**Additional file 2: Table S1.** Signature genes of the HDST breast cancer data.

**Additional file 3: Table S2.** Signature genes of the HDST mouse olfactory bulb data.

**Additional file 4:**

## Data Availability

The DeepLinc pipeline has been deposited in github (https://github.com/xryanglab/DeepLinc) [68] and Zenodo (10.5281/zenodo.6564143) [69]. The source code is released under MIT License (http://opensource.org/licenses). Four published datasets were used for the current study [13, 17, 18], including mouse visual cortex profiled by seqFISH [17, 70], preoptic region of the mouse hypothalamus by MERFISH [18, 71], mouse olfactory bulb and human breast cancer by HDST [13, 72].
