# Supplementary material for: De novo reconstruction of cell interaction landscapes from single-cell spatial transcriptome data with DeepLinc
Source: Genome Biol. 2022 Jun 3;23:124. doi: 10.1186/s13059-022-02692-0 (PMC9164488; doi:10.1186/s13059-022-02692-0)
Supplement: Supplementary file 1 — Additional file 1: Figures S1-S10 [file 13059_2022_2692_MOESM1_ESM.docx]

# Supplementary Figures

*De novo* reconstruction of cell interaction landscapes from single-cell spatial transcriptome data with DeepLinc

Runze Li^1^, Xuerui Yang^1^

^1^ MOE Key Laboratory of Bioinformatics, Center for Synthetic & Systems Biology, School of Life Sciences, Tsinghua University, Beijing, China

Correspondence: Xuerui Yang, School of Life Sciences, Tsinghua University, Beijing 100084, China. Tel: 86-10-62783943. Email: yangxuerui@tsinghua.edu.cn


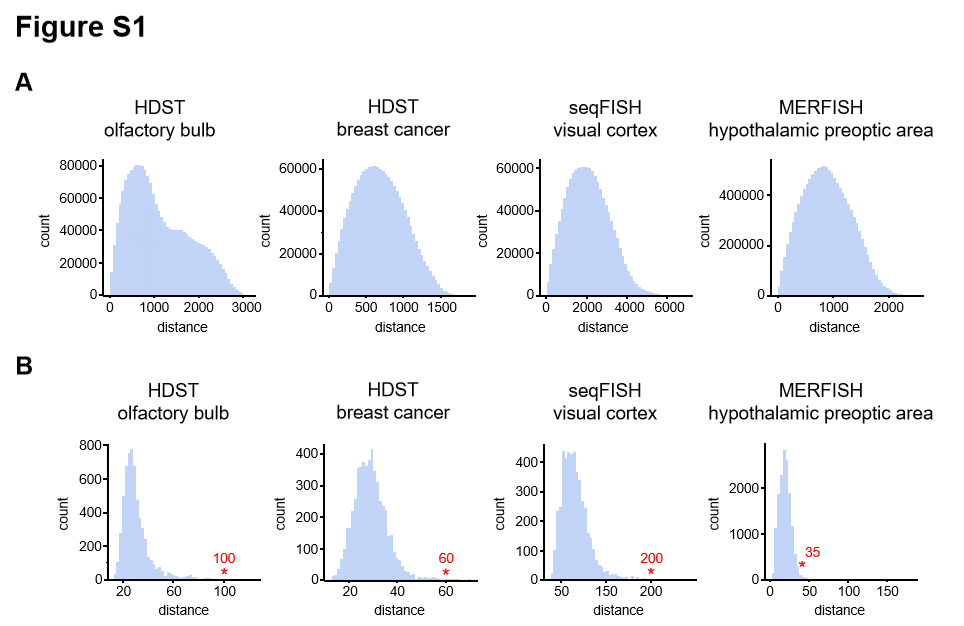


Figure S1. Distribution of the geometric distances between cells.

**(A)** Distribution of the distances between each pair of cells in the 4 datasets.

**(B)** Distribution of the distances between each cell and the 3 closest neighbors. * marks the distance thresholds used to define direct cell-cell contacts.


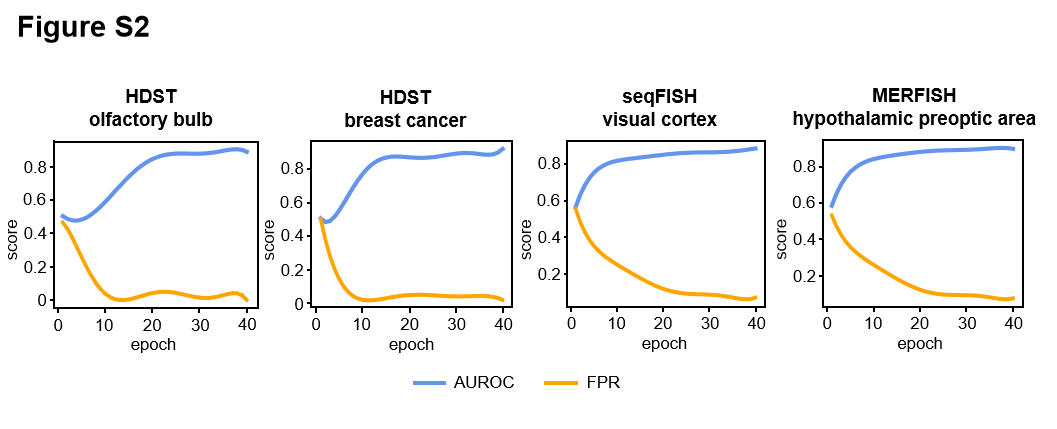


Figure S2. Performance of DeepLinc in reconstructing cell interaction networks.

The levels of AUROC at different training epochs of DeepLinc. The area under the receiver operating characteristic (AUROC) and the false positive rate (FPR) were plotted at different training epochs.


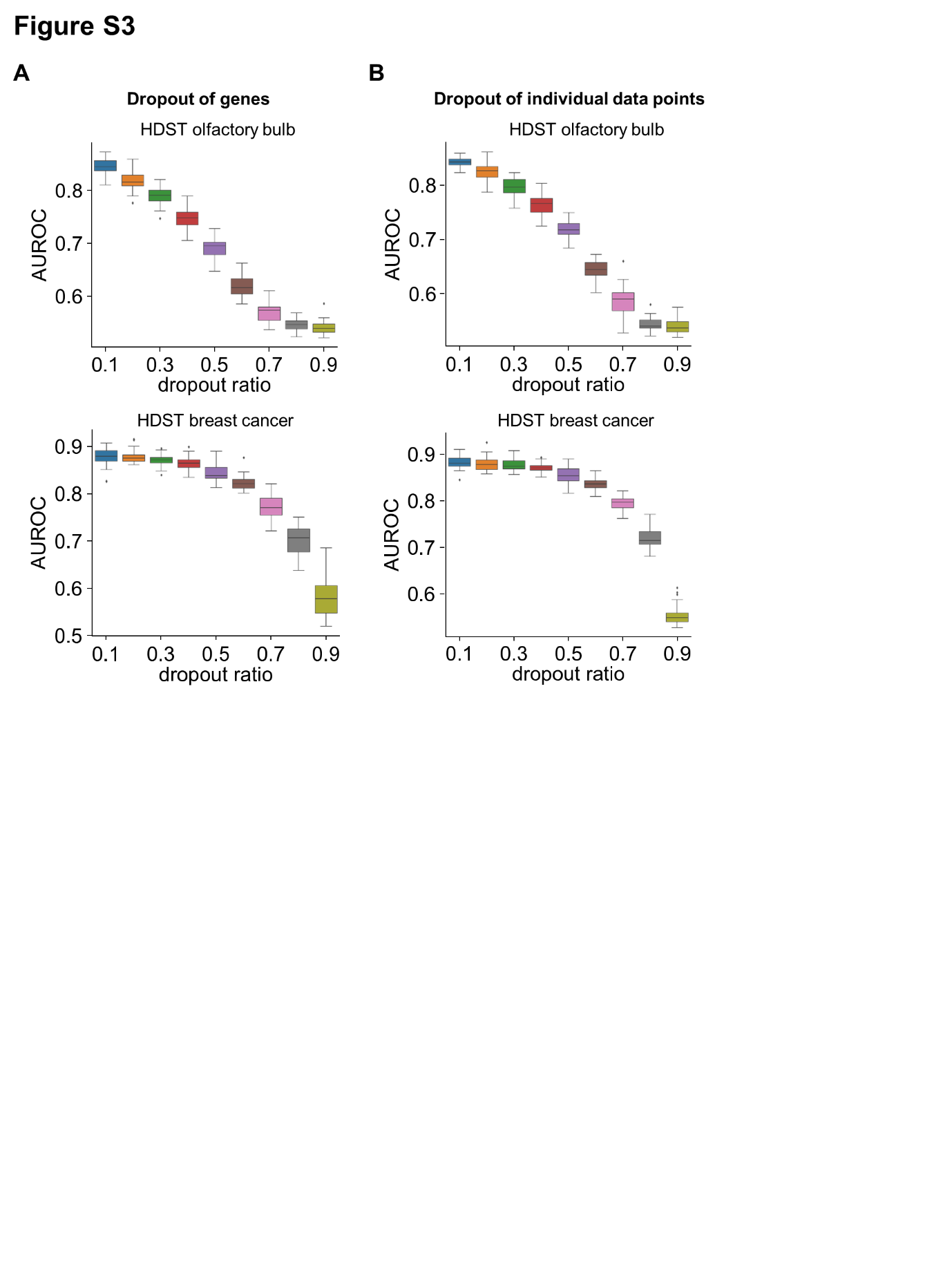


Figure S3. Performance of DeepLinc with different types and ratios of dropouts.

Two different types of dropouts from the original single-cell gene expression data were simulated for testing the performance of DeepLinc. (A) Different percentages of genes were randomly removed and (B) different percentages of the non-zero values were randomly picked and forced to be zero in the whole transcriptome dataset. Different proportions of dropouts were used. This process was repeated 30 times for each proportion of dropout to draw a boxplot.


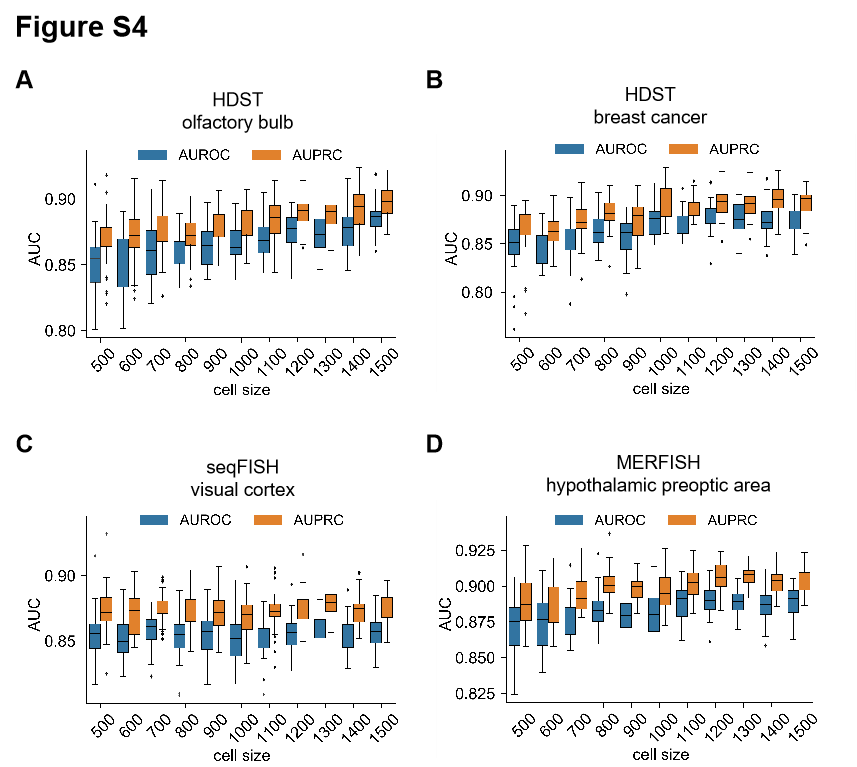


Figure S4. Performance of DeepLinc with different numbers of cells.

Different sizes of tissue regions (different numbers of cells) were randomly selected for reconstruction of cell interaction networks by DeepLinc. For each size, the same procedure was repeated for 30 times, and the testing set was randomly selected each time (training set: testing set=9:1). The area under the receiver operating characteristic (AUROC) were summarized as box plots. For comparison, we adopted the same model structure and number of training iterations for all the tests.


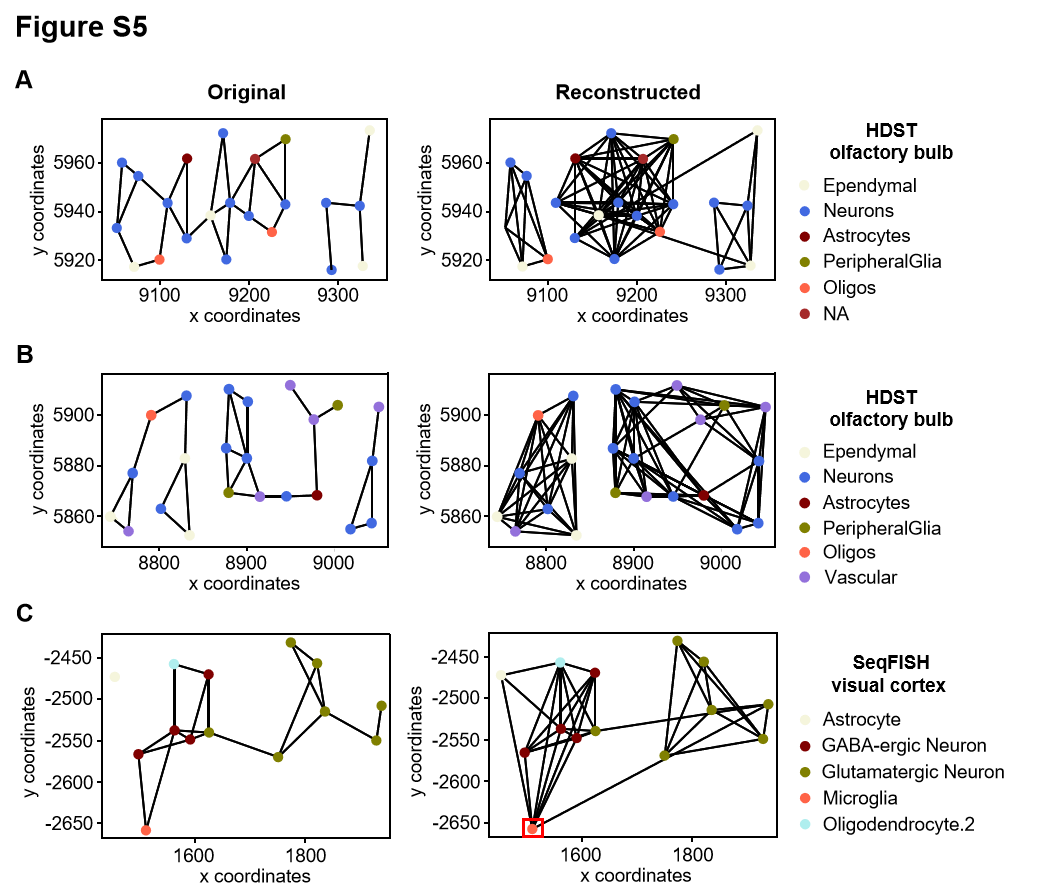


Figure S5. Original and reconstructed cell interaction networks in representative tissue regions.

Representative tissue regions were selected from the HDST data set of olfactory bulb (A, B), seqFISH dataset of visual cortex (C), and MERFISH dataset of hypothalamic preoptic area (D). The red box in panel C indicates a microglia cell with distal interactions with many other cells in the reconstructed networks.


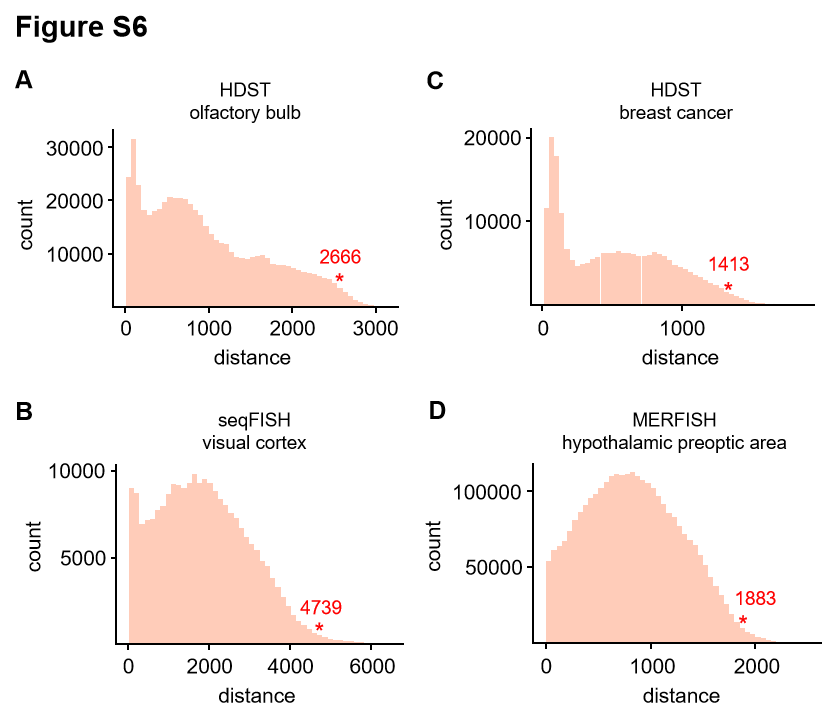


Figure S6. Distance distribution of the interacting cell pairs in the reconstructed cell networks.

Histograms showing the geometric distances between all the interacting cell pairs in the reconstructed cell interaction networks. The distance thresholds used to define distal interactions are marked on the plots.


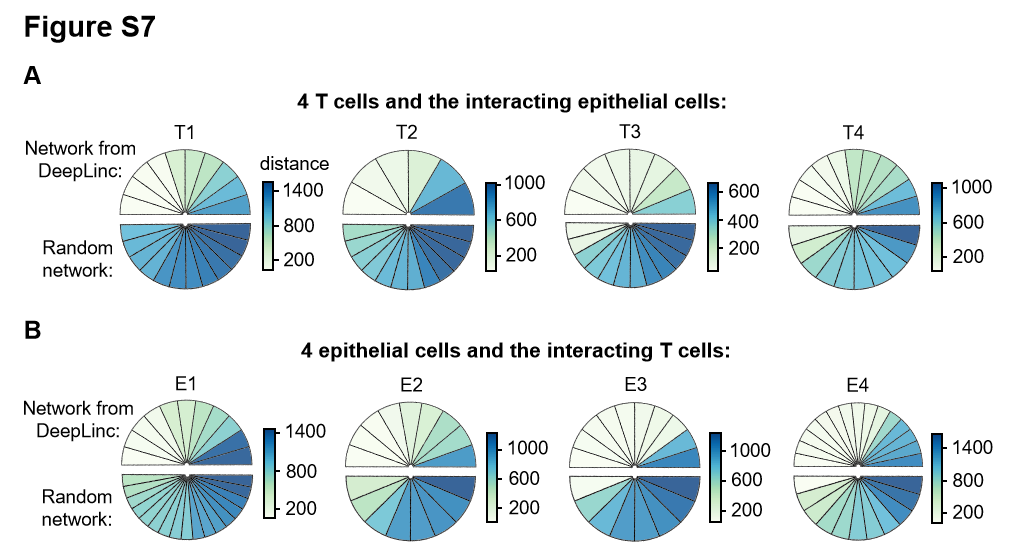


Figure S7. Geometric distances between interacting T cells and epithelial cells in the breast cancer tissue.

4 T cells (A) and 4 epithelial cells (B) were randomly selected in the HDST breast cancer data as examples. Each circular plot represents a T cell (A) or an epithelial cell (B), in which the sectors represent the interacting epithelial cells (A) or T cells (B), respectively. For each circular plot, the interacting cells were drew from the cell interaction network reconstructed by DeepLinc (upper half) or from a random network with the same size (lower half). Each sector was color coded according to the geometric distance between the two cells on the tissue section.


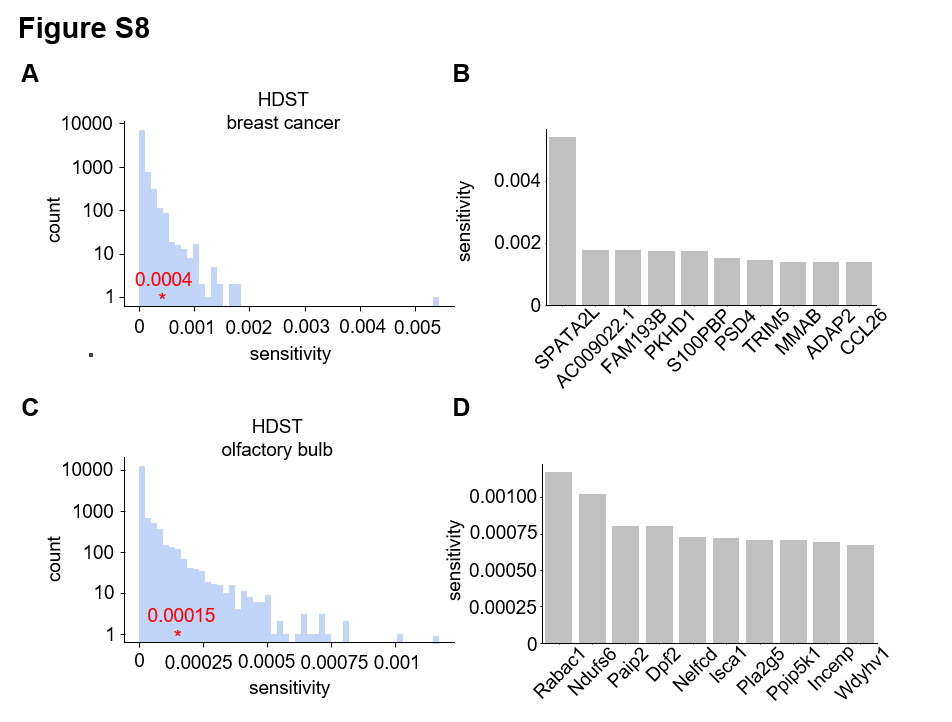


Figure S8. Sensitivity scores of genes in the HDST datasets.

**(A, C)** Distributions of the sensitivity scores of all the genes in the HDST breast cancer (A) or HDST olfactory bulb (C) dataset. Cutoffs of sensitivity score used to identify the signature genes are marked on the plots.

**(B, D)** Top 10 genes with the highest sensitivity scores.


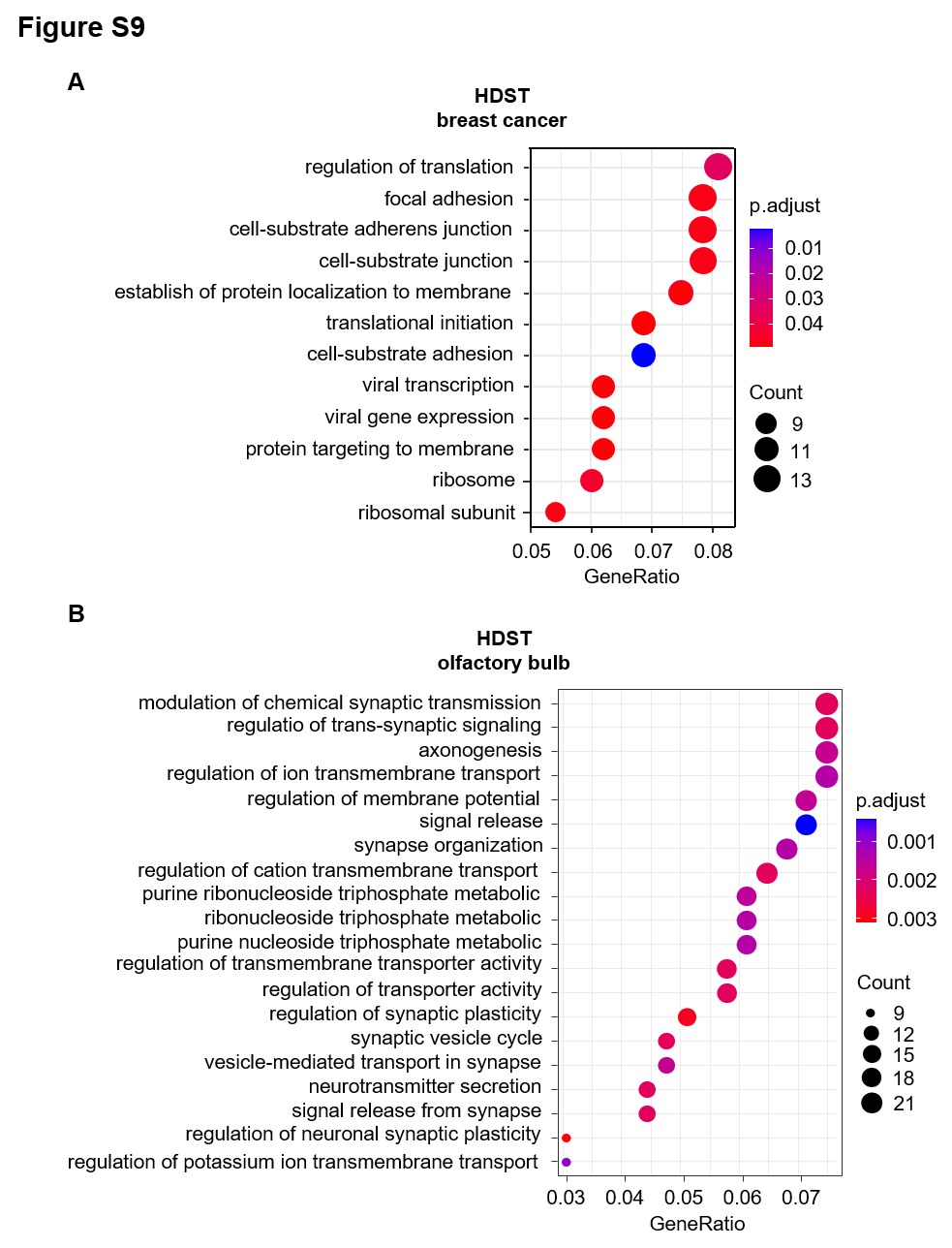


Figure S9. Full lists of the GO categories enriched by the signature genes from the HDST datasets.

For the two high-dimensional datasets of HDST, the biological processes involving the top-ranked genes were obtained through Gene Ontology Enrichment Analysis. All the top-rated GO categories are being provided in (A) for breast cancer and (B) for olfactory bulb.


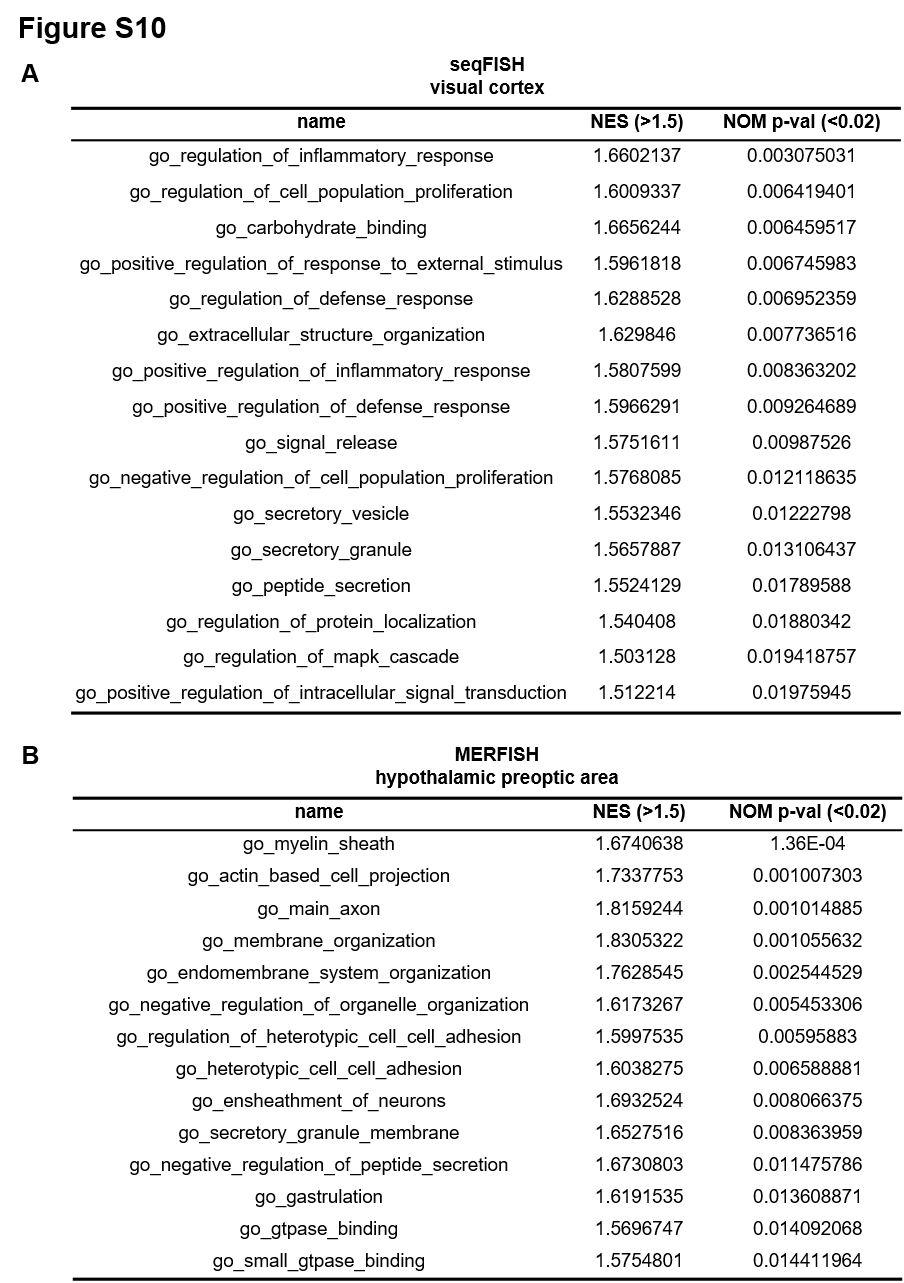


Figure S10. Full lists of enriched biological processes by GSEA analysis in seqFISH and MERFISH datasets.
