# Supplementary material for: De novo reconstruction of cell interaction landscapes from single-cell spatial transcriptome data with DeepLinc
Source: Genome Biol. 2022 Jun 3;23:124. doi: 10.1186/s13059-022-02692-0 (PMC9164488; doi:10.1186/s13059-022-02692-0)
Supplement: Supplementary file 4 — Additional file 4: [file 13059_2022_2692_MOESM4_ESM.docx]

Review History

**First round of review**

**Reviewer 1**

**Were you able to assess all statistics in the manuscript, including the appropriateness of statistical tests used?**

Yes

**Comments to author:**

The paper presents a graph neural network (GNN) for inferring cell neighborhoods in spatial transcriptomics data. The method takes as input a graph structure in which every cell is connected to its three closest cells in space, and the expression matrix for each cell and outputs predictions about the spatial relationship of any pair of cells (i.e. tries to predict edges in the graph defined by the authors). The authors perform several technical evaluations, discuss some of the biological implications of the edges identified for non neighboring cells and some of the genes that are determined to be important for the prediction task.

While the method itself is of interest (combination of variational encoder and adversarial networks) as the authors note this idea is not new and based on prior work. The main claim of the paper though is problematic. I am not convinced that the method indeed learns interactions between cells. It does learn spatial organization features (so which cells are neighbors) but that does not necessarily have anything to do with interactions. Not all neighboring cells interact and not all interacting cells are neighbors. The only relevant evidence that the method indeed infers interactions is the analysis of top genes (Figure 4) that lists some signaling genes as important and some categories as significant. However, as I note below much more information is required to assess if these are indeed the features that drive the predictions and what they mean.

Major comments:

1. The paper, including title, abstract and text keeps talking about inferring interactions. But the method does not attempt to infer interactions at all. It only tries to infer spatial neighborhoods. Whether these are based on interactions is not clear so I would completely revise the description in all these places and throughout the paper to make it clear what you are using and what you are predicting.

2. The authors claim to have good results in predicting hidden edges. But in reality, for each cell there are only 3 real edges and thousands of non edges. If the authors would have used this proportion in their classification the results would be terrible (i.e. for almost all cells the correct edges would not be anywhere near the top 10 or even top 100). Instead, the authors balance the test set (a random 3 edges and the real 3 edges for each cell). This, of course, makes the prediction very easy since random edges are very unlikely to resemble a real neighborhood. But its not really clear what it shows for the overall predictions. What would be the results if you kept the true real and not real edges for each cell?

3. Much of the results can be explained by clustering. i.e. perform spatial clustering, as several other methods do and then predict neighbors from a test set based on the cluster the cell is in. Did you try this approach?

4. Why use Gaussian noise for gene expression in single cells? The main issue in single cells is dropout. The noise model should take that into account and attempt to remove genes rather than just adding Gaussian noise.

5. Where is the full list of 205 genes identified as significant for the classifier? I could not find it in the supplement. Are the GO categories shown in Figure 4 comprehensive? Are these the only categories for these 205 genes? Results for the FISH data is less interesting since there are very few genes to begin with so the list is biased. But it is important to see the full list for the 2 sequencing based dataset and to test whether they are indeed dominated by signaling genes. If the top categories are not signaling then the method simply identifies clusters (not necessarily homogenous in terms of cell types) in 2D. This is of interest, but does not imply interactions.

6. The ability to infer long term interactions based on the method (Figure 3 and text) is also questionable. It seems to me that the predictions are mainly based on the spatial proximity training data and generalize form that. Can you show examples of things that are predicted to interact only long range and are not close together in the 2D images?

**Reviewer 2**

**Were you able to assess all statistics in the manuscript, including the appropriateness of statistical tests used?**

No

**Comments to author:**

In this manuscript, the authors developed a deep generative model based on VGAE, named DeepLinc, for encoding cell-cell interaction features from spatial single-cell transcriptome data and eventually regenerating full cell-cell interaction landscapes. DeepLinc was designed to directly uncover the cell-cell interactions that shape the tissue organization and define tissue physiological functions. The most advantage of DeepLinc is that it does not depend on prior knowledge of cell types, ligand-receptor pairs, or cell interaction mechanisms. The authors tested their model on 4 spatially resolved single-cell transcriptomes and demonstrated the high efficiency of DeepLinc in learning from imperfect and incomplete spatial transcriptome data, filtering false interactions, and imputing missing distal and proximal interactions. The manuscript scientifically sounds and was overall well organized. However, the following concerns may need attention.

Major:

1. It's may out of the scope of this work but worth a try that use DeepLinc to more different datasets from distinct techniques, such as ST/10X Visium and Slide-seq. Especially for the most widely and commercially used 10X platform, the resolution is not exactly at a single-cell level rather 2-10 cells per spot. It would be of more interest whether and how the DeepLinc framework will be beneficial to this kind of spatial transcriptome by deconvoluting the multi-cell spots for cell-cell interaction network reconstruction.

2. More clarifications are needed for why only the 3 closet neighbors are chosen to define the direct contacts. If it's not from the biological and technological basis, a series of different numbers of neighbors should be tested before choosing a specific number for downstream analysis.

3. An additional concern related to the above one: geometric proximities do not always imply functional interactions between cells and vice versa. Although the authors tested DeepLinc in recovering the cell-cell interaction networks from artificially removed or added edges, it's worth noting that the predefined network by choosing 3 closet neighbors per se is not the ground truth, which also contains false and missing edges. DeepLinc is therefore expected to recover the missing edges not covered by "3 closet neighbors" (biologically false negatives) or remove the false edge only based on geometric proximity (biologically false positive). So, more benchmarking tests are needed in this regard, for which the additional possible examples are to use the known ligand-receptor-based cell-cell interactions as the gold standard, which will reflect the cases that geometric neighbor cells may not interact and distal cells may interact by cytokine transition. Doing this will help a lot to demonstrate that DeepLinc will benefit the reconstruction of cell-cell interaction landscapes in biological regard (embedded in the gene expression profiles), including both the proximal and distal communications.

4. The section "Reconstructed cell-cell interaction landscapes" partially resolved the concerns in above comment 3. However, in Figure 3, I'd prefer to include the network plots of at least one dataset as an example in Figure S3 for a more intuitive impression to the readers, rather than just putting Figure 3 with all statistic results.

Minor:

1. It would be better for Figures S1 and S4 to give the exact thresholds in the plots, except for using * marks.

2. On page 5 line 44 and thereafter, the term "dot production" is usually written in "dot product" as terminology in mathematics. And in the formulation, the dot product should be denoted using a dot (·) rather than an asterisk (*) such as in Figure 1A.

3. On the GitHub repository, to make the codes friendlier to users, it's suggested to add more information and codes on how to prepare the input files from the commonly used ST data. Currently, there's no information on the detailed format of the input file, except for test data for seqFISH.

**Authors Response**

**Point-by-point responses to the reviewers’ comments:**

*General responses to the Reviewers’ Comments*

*We would like to thank the reviewers for their time and efforts in reviewing our manuscript. The thoughtful suggestions and comments have greatly helped us improve the manuscript. We now have added a series of new analyses and more discussions to fully address the questions. Please refer to the following point-by-point responses for the details.*Reviewer #1:

The paper presents a graph neural network (GNN) for inferring cell neighborhoods in spatial transcriptomics data. The method takes as input a graph structure in which every cell is connected to its three closest cells in space, and the expression matrix for each cell and outputs predictions about the spatial relationship of any pair of cells (i.e. tries to predict edges in the graph defined by the authors). The authors perform several technical evaluations, discuss some of the biological implications of the edges identified for non neighboring cells and some of the genes that are determined to be important for the prediction task.

While the method itself is of interest (combination of variational encoder and adversarial networks) as the authors note this idea is not new and based on prior work. The main claim of the paper though is problematic. I am not convinced that the method indeed learns interactions between cells. It does learn spatial organization features (so which cells are neighbors) but that does not necessarily have anything to do with interactions. Not all neighboring cells interact and not all interacting cells are neighbors. The only relevant evidence that the method indeed infers interactions is the analysis of top genes (Figure 4) that lists some signaling genes as important and some categories as significant. However, as I note below much more information is required to assess if these are indeed the features that drive the predictions and what they mean.

-*Response: We thank the reviewer for the thoughtful comments. There have been a number of methods to infer cell-cell interactions from single-cell transcriptomic profiles (Browaeys et al., 2020; Cang and Nie, 2020; Costa et al., 2018; Dries et al., 2019; Efremova et al., 2020; Kumar et al., 2018; Nol et al., 2020; Richelle et al., 2019). Most of these methods rely on pre-defined features such as the known ligand-receptor pairs and cofactors (Efremova et al., 2020; Nol et al., 2020), downstream signaling gene products and gene regulatory networks (Browaeys et al., 2020; Cang and Nie, 2020), and the intracellular metabolites and signaling molecules (Richelle et al., 2019), for the definition of cell-cell interactions. However, quantitative profiles of these features are very limited for the spatially resolved single-cell transcriptomic data, due to the high drop-out rates and high noise levels. To the best of our knowledge, DeepLinc is the first of its kind for de novo reconstruction of cell-cell interaction networks from spatially resolved single-cell transcriptome profiles, in a more unbiased manner independent of prior knowledge such as ligand-receptor pairs.

It is well recognized that single-cell transcriptome profiles represent both the driving force and consequences of cell-cell interaction landscapes. DeepLinc further assumes that the neighboring cells close to each other should be enriched by cell-cell interactions, which are reflected by the transcriptome profiles of the cells. The pipeline of DeepLinc combining a VGAE and an adversarial network was then designed for mining of the latent associations between the cell-cell interactions and the transcriptome profiles of the neighboring cells, which were then used to filter out the non-interacting neighboring cells and infer the distal interactions between non-neighboring cells.

As the reviewer has pointed out, the functional annotations of the top signature genes suggest effective learning and prediction by DeepLinc. We have added further information to address the reviewer’s question here. Please refer to the following response to the comment #5.*Major comments:
1. The paper, including title, abstract and text keeps talking about inferring interactions. But the method does not attempt to infer interactions at all. It only tries to infer spatial neighborhoods. Whether these are based on interactions is not clear so I would completely revise the description in all these places and throughout the paper to make it clear what you are using and what you are predicting.

*-Response: We are sorry for not making the strategy of DeepLinc clearer. DeepLinc was indeed designed to infer cell-cell interactions, including both the local interactions between neighboring cells and the distal interactions.

Specifically, DeepLinc assumes that the neighboring cells should be much more likely to have some types of interactions than randomly picked non-neighboring cells that are far away from each other. Therefore, for a particular tissue region, the neighboring cell pairs comprise an incomplete and potentially noisy observation of a subset of the full cell-cell interaction network. It is well recognized that single-cell transcriptome profiles represent both the driving force and consequences of cell-cell interaction landscapes. Therefore, the main task of DeepLinc was to learn from the incomplete and noisy set of cell-cell interactions, extract the latent features related to the interactions, and finally, regenerate a more unbiased and complete landscape of cell-cell interactions, which would include both proximal and distal interactions.

As shown in Figs. 4 and S5, DeepLinc recovered great numbers of distal interactions, and meanwhile, DeepLinc removed some of the predefined proximal interactions between neighboring cells. Indeed, based on a wide array of tests with real and simulated data, DeepLinc demonstrated high efficiency in learning from imperfect and incomplete spatial transcriptome data, filtering false interactions, and inferring missing distal and proximal interactions (Figs. 2-4). The reconstructed full networks of cell interactions exhibited high physiological relevance. Therefore, DeepLinc serves as a tool for de novo reconstruction of cell interaction networks in a more comprehensive manner by learning from the spatial neighborhoods and the transcriptome features of single-cells.

As the reviewer has pointed out, our further interrogation of the pipeline revealed signature genes that are potentially involved in shaping the cell interaction landscapes. These genes are indeed highly enriched by the processes related to distal and proximal cell-cell interactions, indicating effective learning and prediction by DeepLinc (Fig. 5). This also proves the validity of the basic assumptions of DeepLinc.*
*As suggested, we have revised the text of the manuscript to better describe the assumptions, the rational, and the purpose of DeepLinc in the sections of Introduction (Page 4), Results (Page 5), Discussion (Page 22) and Methods (Page 24).*2. The authors claim to have good results in predicting hidden edges. But in reality, for each cell there are only 3 real edges and thousands of non edges. If the authors would have used this proportion in their classification the results would be terrible (i.e. for almost all cells the correct edges would not be anywhere near the top 10 or even top 100).
Instead, the authors balance the test set (a random 3 edges and the real 3 edges for each cell). This, of course, makes the prediction very easy since random edges are very unlikely to resemble a real neighborhood. But its not really clear what it shows for the overall predictions. What would be the results if you kept the true real and not real edges for each cell?

*-Response: The reviewer has raised a very valid question. We used the testing sets simply to benchmark the performance of DeepLinc in differentiating the positive and negative edges. In response to the reviewer’s comment, we generated new imbalanced testing sets with high negative-to-positive ratios (e.g., 100:1) to evaluate the capability of DeepLinc in identifying the true positive interactions aside from a much larger pool of true negatives. As shown in Figs. 2A and S2, with the training processes, DeepLinc reached high sensitivity and accuracy with AUROC above 0.85 and false positive rate (FPR) below 5% for all of the 4 datasets. This shows that DeepLinc could efficiently adapt to the imbalanced dataset, which, as the reviewer has pointed out, is critical when dealing with real data.

Furthermore, we repeated all the previously done benchmarks for DeepLinc (Fig. 2), but with the imbalanced testing sets. These include the tolerance of DeepLinc to gene expression noise (Fig. 2A), the imputation of missing interactions (Fig. 2B), and the capability of denoising with random false interactions (Fig. 2C). In brief, the performances of DeepLinc were consistently well with the imbalanced testing sets. Specifically, DeepLinc was still quite robust to random noise in gene expression data (Fig. 2A). DeepLinc also exhibited high accuracy in imputing the missing positive interactions. For example, when half of the interactions were lost in the input, DeepLinc still managed to recover these missing edges with 65–75% AUROC (Fig. 2B). Lastly, DeepLinc can still nicely distinguish the originally nonexistent edges and the real pre-existing edges with random false interactions (Fig. 2C). All the previous plots with the balanced testing sets have been replaced by the new benchmarking results with the imbalanced sets (Figs. 2 and S2).*3. Much of the results can be explained by clustering. i.e. perform spatial clustering, as several other methods do and then predict neighbors from a test set based on the cluster the cell is in. Did you try this approach?

*-Response: As discussed above, DeepLinc was not designed to just predict spatial neighborhoods. Instead, DeepLinc learns from the spatial neighborhoods and the transcriptome features of single-cells, in order to infer a more complete set of interactions between either neighboring or non-neighboring cells. The distal interactions predicted by DeepLinc would not be recovered from spatial clusters.

It is worth noting that the latent features of spatial transcriptome profiles learned by DeepLinc can also be used for spatial clustering (Fig. 6). The spatially coded clusters indeed showed physiologically relevant patterns. As expected, the interactions predicted by DeepLinc are certainly not limited just within these clusters.

Therefore, being positioned for de novo reconstruction of the cell interaction landscapes including both distal and proximal interactions, DeepLinc is a new type of method and cannot be simply replaced by the methods of spatial clustering.*
4. Why use Gaussian noise for gene expression in single cells? The main issue in single cells is dropout. The noise model should take that into account and attempt to remove genes rather than just adding Gaussian noise.

*-Response: We thank the reviewer for the very thoughtful suggestion. Indeed, single-cell spatial transcriptome data is largely suffering from high sparsity due to dropouts. We now have generated new models of noise to simulate different types of dropouts.
1) Dropouts of genes: From the whole transcriptome dataset, different percentages of genes were randomly removed.
2) Dropouts of individual data points: In the whole transcriptome dataset, different percentages of the non-zero values were randomly picked and forced to be zero.
As shown in Fig. S3, under both of these scenarios, DeepLinc showed high tolerance to the noise from dropouts of genes or individual data points. In general, the performances of DeepLinc were still fairly good with the dropout ratio of 50%. As well expected, more severe dropouts would strongly reduce the accuracy of DeepLinc. In summary, these new tests confirm the advantage of DeepLinc in extracting the latent information of cell-cell interactions from the highly sparse and noisy spatial transcriptome profiles. These results have been provided and discussed on page 9.*5. Where is the full list of 205 genes identified as significant for the classifier? I could not find it in the supplement. Are the GO categories shown in Figure 4 comprehensive? Are these the only categories for these 205 genes? Results for the FISH data is less interesting since there are very few genes to begin with so the list is biased. But it is important to see the full list for the 2 sequencing based dataset and to test whether they are indeed dominated by signaling genes. If the top categories are not signaling then the method simply identifies clusters (not necessarily homogenous in terms of cell types) in 2D. This is of interest, but does not imply interactions.

*-Response: Full lists of the signature genes have been provided in Supplementary Table S1 and S2.

The GO categories shown in Fig. 5 were not the full list. The duplicated categories, categories that are too general or potentially misleading have been removed to save space. To answer the reviewer’s question, all the top-ranked GO categories above statistical thresholds from the two HDST datasets (breast cancer and olfactory bulb) are now supplied in Fig. S9. As shown by the complete lists, the signature genes are overwhelmingly enriched by the processes related to several types of cell interactions in the cancer and nervous system, which are shown in the short lists in Fig. 5.

We agree with the reviewer that the signature genes from the two FISH datasets were too few for meaningful GO enrichment analyses. Therefore, we used a different strategy, i.e., gene set enrichment analysis (GSEA), to evaluate the processes involving more of the top-rated signature genes. The full lists are also provided in Fig. S10, which again are dominated by the processes known to be important for shaping the cell interaction landscapes.

Taken together, the results above indicate that DeepLinc indeed has learned key features related to cell interaction landscapes. Furthermore, the signature gene lists also shed lights on the biological processes that play key roles in defining the different types of cell interaction patterns under specific tissue contexts.*
6. The ability to infer long term interactions based on the method (Figure 3 and text) is also questionable. It seems to me that the predictions are mainly based on the spatial proximity training data and generalize form that. Can you show examples of things that are predicted to interact only long range and are not close together in the 2D images?

*-Response: We are sorry for not making this clearer. Examples of the reconstructed networks were provided in Fig. S3 in the original manuscript, which included both local and distal interactions. They now have been moved into the main figures (Fig. 3E) and Fig. S5 in the revised manuscript. Specifically, these figures of representative tissue regions illustrated the reconstructed cell interaction networks, which showed very different patterns compared to the original neighborhood networks. Distal interactions between non-neighboring cells have been reinstated by DeepLinc after reconstruction. For example, the red boxes in Fig. 3E and S5C marked microglia connecting with many other cells via distal interactions, which is well supported by the wide range of interactions between microglia and other cell types in the CNS (Prinz et al., 2019).*
*As suggested by the reviewer, we have added more examples to emphasize the distal interactions inferred by DeepLinc (Fig. 4E-G). Specifically, with the seqFISH data of visual cortex, DeepLinc recovered distal interactions highly enriched by neurons (Fig. 4A). An example is now provided in Fig. 4E to show the interactions between a glutamatergic neuron and other GABA-ergic neurons. Such distal interactions certainly cannot be inferred simply from spatial clustering. In other examples, with the HDST data of breast cancer, DeepLinc recovered distal interactions enriched between epithelial and stromal cells and between T cells (Fig. 4D). As examples, the distal interactions between an epithelial cell and multiple stromal cells and between a T cell and other T cells have been illustrated in Fig. 4F, G.*

Reviewer #2:

In this manuscript, the authors developed a deep generative model based on VGAE, named DeepLinc, for encoding cell-cell interaction features from spatial single-cell transcriptome data and eventually regenerating full cell-cell interaction landscapes. DeepLinc was designed to directly uncover the cell-cell interactions that shape the tissue organization and define tissue physiological functions. The most advantage of DeepLinc is that it does not depend on prior knowledge of cell types, ligand-receptor pairs, or cell interaction mechanisms. The authors tested their model on 4 spatially resolved single-cell transcriptomes and demonstrated the high efficiency of DeepLinc in learning from imperfect and incomplete spatial transcriptome data, filtering false interactions, and imputing missing distal and proximal interactions. The manuscript scientifically sounds and was overall well organized. However, the following concerns may need attention.

*-Response: We thank the reviewer for the positive remarks and thoughtful suggestions. Following are our detailed responses to the comments.*Major:
1. It's may out of the scope of this work but worth a try that use DeepLinc to more different datasets from distinct techniques, such as ST/10X Visium and Slide-seq. Especially for the most widely and commercially used 10X platform, the resolution is not exactly at a single-cell level rather 2-10 cells per spot. It would be of more interest whether and how the DeepLinc framework will be beneficial to this kind of spatial transcriptome by deconvoluting the multi-cell spots for cell-cell interaction network reconstruction.

*-Response: The current version of DeepLinc requires spatial transcriptome profiles at single-cell resolution. As the reviewer has pointed out, a series of spatial transcriptome profiling techniques, for example ST/10X Visium and Slide-seq, have generated data of multi-cellular tissue spots, rather than individual cells. Several tools have been proposed for the task of deconvolution (Andersson et al., 2020; Cable et al., 2021; Danaher et al., 2022; Elosua-Bayes et al., 2021; Song and Su, 2021; Sun et al., 2022; Yang et al., 2021). However, all these existing tools were aimed to deconvoluting for the proportions of different cell types within each spot based on the accumulative gene expression profiles. None of them was designed to further infer the gene expression profile of each single-cell from the data of multi-cell spots. Despite our best effort, we could not reinstate the single-cell spatial transcriptome profiles from the ST/10X Visium datasets. There is no such data publicly available either. Therefore, although we agree with the reviewer that it would be nice to test the performance of DeepLinc on the spatial transcriptome data at multi-cell resolution, we could not figure out a reliable deconvolution method to obtain single-cell spatial transcriptomes as inputs of DeepLinc.

Nevertheless, technical advances in the future would for sure generate more of the spatial transcriptome profiles at single-cell resolution. Given the consistently robust performances of DeepLinc with the 4 different datasets in our study, we have no reason to doubt that DeepLinc will not perform the same, if not better, on future data with likely higher quality.*
2. More clarifications are needed for why only the 3 closet neighbors are chosen to define the direct contacts. If it's not from the biological and technological basis, a series of different numbers of neighbors should be tested before choosing a specific number for downstream analysis.

*-Response: We are sorry for not making this clearer. DeepLinc uses neighboring cells with direct contacts as the positive set for learning the transcriptome features related to cell-cell interactions. From a general biological point of view, we think it is reasonable to assume that in a solid tissue, most of the cells in 2-D could directly contact with 3 or more other cells. From a technical point of view, for the strategies of machine learning, it is critical to minimize the potential false positives in the predefined positive sets for training. Therefore, for each cell, we only used the 3 nearest neighbors to define direct contacts, which we believe is a balanced choice generating enough number of direct contacts for training the DeepLinc pipeline, and at the same time, ensuring few false positives to contaminate the positive training set.

Nevertheless, the performances of DeepLinc were quite consistent when using the nearest 2, 3, 4, or 5 neighbors as the positive sets of direct contacts. This suggests high adaptability of DeepLinc to different sizes of positive sets of direct contacts, which may vary in the rates of false positives. This again illustrates the learning efficiency of DeepLinc and its robustness to intrinsic data noise. Indeed, as shown in Fig. 2 B, C and S4, DeepLinc has demonstrated high tolerance to missing and fake edges and consistent performances with fewer input data. Therefore, it appears that the predefined networks composed of 3 closest neighbors have provided enough information for DeepLinc to mine for the latent association between intrinsic transcriptome features and cell-cell interactions.*3. An additional concern related to the above one: geometric proximities do not always imply functional interactions between cells and vice versa.
Although the authors tested DeepLinc in recovering the cell-cell interaction networks from artificially removed or added edges, it's worth noting that the predefined network by choosing 3 closet neighbors per se is not the ground truth, which also contains false and missing edges. DeepLinc is therefore expected to recover the missing edges not covered by "3 closet neighbors" (biologically false negatives) or remove the false edge only based on geometric proximity (biologically false positive). So, more benchmarking tests are needed in this regard, for which the additional possible examples are to use the known ligand-receptor-based cell-cell interactions as the gold standard, which will reflect the cases that geometric neighbor cells may not interact and distal cells may interact by cytokine transition. Doing this will help a lot to demonstrate that DeepLinc will benefit the reconstruction of cell-cell interaction landscapes in biological regard (embedded in the gene expression profiles), including both the proximal and distal communications.

*-Response: We totally agree with the reviewer that the predefined network of cell neighborhoods contains false and missing edges. In fact, DeepLinc assumes that the neighboring cells should be much more likely to have some types of interactions than randomly picked non-neighboring cells that are far away from each other. Therefore, for a particular tissue region, the neighboring cell pairs comprise an incomplete and potentially noisy observation of a subset of the full cell-cell interaction network. It is well recognized that single-cell transcriptome profiles represent both the driving force and consequences of cell-cell interaction landscapes. Therefore, as the reviewer has pointed out, the main task of DeepLinc was to learn from the incomplete and noisy set of cell-cell interactions, extract the latent features related to the interactions, and finally, regenerate a more unbiased and complete landscape of cell-cell interactions, which would include both proximal and distal interactions.*
*As shown in Fig. 4 and S5, DeepLinc recovered great numbers of distal interactions, and meanwhile, DeepLinc removed some of the predefined proximal interactions between neighboring cells. Indeed, based on a wide array of tests with real and simulated data, DeepLinc demonstrated high efficiency in learning from imperfect and incomplete spatial transcriptome data, filtering false interactions, and inferring missing distal and proximal interactions (Figs. 2-4). The reconstructed full networks of cell interactions exhibited high physiological relevance.

We agree that the distal cell-cell interactions based on ligand-receptor pairs would serve as an insightful resource for another benchmark of DeepLinc. However, quantitative profiles of the known ligand and receptor genes are very limited for the spatially resolved single-cell transcriptomic data, due to the high drop-out rates and high noise levels. Most of the ligand or receptor genes were not detected in enough numbers of single-cells. For example, from a commonly used resource composed of 2,558 annotated ligand-receptor pairs (Ramilowski et al., 2015), only 105 ligands and 130 receptors were expressed with a non-zero value in at least one cell of the HDST olfactory bulb data. The most frequently detected ligand and receptor genes were merely expressed in 48 and 17 cells, respectively. Fewer than 5 pairs of ligand and receptor were detected in more than 25 cell pairs. A maximum of 2 ligand-receptor pairs were detected simultaneously in any pair of cells, and this was observed only in 20 cell pairs. Such extremely* *low coverages of the ligand and receptor genes were also seen in the HDST breast cancer data. As expected, for the two low-dimensional datasets (seqFISH and MERFISH), the coverages of ligands and receptors were much worse, with only 2 and 18 ligand-receptor pairs detected in at least one pair of cells, respectively.

Therefore, the extremely low coverages of the known ligands and receptors in the current single-cell spatial transcriptome data have prohibited inferring of cell-cell interactions based on the annotated ligand-receptor pairs. This necessitates de novo methods, such as DeepLinc, for reconstruction of cell-cell interaction networks in a more unbiased manner independent of prior knowledge such as ligand-receptor pairs.*
3. The section "Reconstructed cell-cell interaction landscapes" partially resolved the concerns in above comment 3. However, in Figure 3, I'd prefer to include the network plots of at least one dataset as an example in Figure S3 for a more intuitive impression to the readers, rather than just putting Figure 3 with all statistic results.

*-Response: We thank the reviewer for the suggestion. We have moved Fig. S3D into the main figures (Fig. 3E) of the revised manuscript. We have also added more examples to emphasize the distal interactions inferred by DeepLinc (Fig. 4E-G).*

Minor:
1. It would be better for Figures S1 and S4 to give the exact thresholds in the plots, except for using * marks.

*-Response: As suggested, the exact thresholds have been provided on the plots in Fig. S1 and S6.*2. On page 5 line 44 and thereafter, the term "dot production" is usually written in "dot product" as terminology in mathematics. And in the formulation, the dot product should be denoted using a dot (·) rather than an asterisk (*) such as in Figure 1A.

*-Response: We thank the reviewer for pointing out these errors. They have been corrected in the revised text and figure.*3. On the GitHub repository, to make the codes friendlier to users, it's suggested to add more information and codes on how to prepare the input files from the commonly used ST data. Currently, there's no information on the detailed format of the input file, except for test data for seqFISH.

*-Response: We thank the reviewer for the reminder. We have added a detailed description of the input files, the full datasets, and a demo for running DeepLinc into the GitHub repository.


References
Andersson, A., Bergenstråhle, J., Asp, M., Bergenstråhle, L., Jurek, A., Fernández Navarro, J., and Lundeberg, J. (2020). Single-cell and spatial transcriptomics enables probabilistic inference of cell type topography. Communications Biology 3, 565.
Browaeys, R., Saelens, W., and Saeys, Y. (2020). NicheNet: modeling intercellular communication by linking ligands to target genes. Nat Methods 17, 159-162.
Cable, D.M., Murray, E., Zou, L.S., Goeva, A., Macosko, E.Z., Chen, F., and Irizarry, R.A. (2021). Robust decomposition of cell type mixtures in spatial transcriptomics. Nature Biotechnology.
Cang, Z., and Nie, Q. (2020). Inferring spatial and signaling relationships between cells from single cell transcriptomic data. Nat Commun 11, 2084.
Costa, A., Kieffer, Y., Scholer-Dahirel, A., Pelon, F., Bourachot, B., Cardon, M., Sirven, P., Magagna, I., Fuhrmann, L., Bernard, C., et al. (2018). Fibroblast Heterogeneity and Immunosuppressive Environment in Human Breast Cancer. Cancer Cell 33, 463-479 e410.
Danaher, P., Kim, Y., Nelson, B., Griswold, M., Yang, Z., Piazza, E., and Beechem, J.M. (2022). Advances in mixed cell deconvolution enable quantification of cell types in spatial transcriptomic data. Nature Communications 13, 385.
Dries, R., Zhu, Q., Dong, R., Linus Eng, C.-H., Li, H., Liu, K., Fu, Y., Zhao, T., Sarkar, A., Bao, F., et al. (2019). Giotto, a pipeline for integrative analysis and visualization of single-cell spatial transcriptomic data. bioRxiv.
Efremova, M., Vento-Tormo, M., Teichmann, S.A., and Vento-Tormo, R. (2020). CellPhoneDB: inferring cell–cell communication from combined expression of multi-subunit ligand–receptor complexes. Nat Protoc 15.
Elosua-Bayes, M., Nieto, P., Mereu, E., Gut, I., and Heyn, H. (2021). SPOTlight: seeded NMF regression to deconvolute spatial transcriptomics spots with single-cell transcriptomes. Nucleic Acids Research 49, e50-e50.
Kumar, M.P., Du, J., Lagoudas, G., Jiao, Y., Sawyer, A., Drummond, D.C., Lauffenburger, D.A., and Raue, A. (2018). Analysis of Single-Cell RNA-Seq Identifies Cell-Cell Communication Associated with Tumor Characteristics. Cell Rep 25, 1458-1468 e1454.
Nol, F., Massenet-Regad, L., Carmi-Levy, I., Cappuccio, A., and Soumelis, V. (2020). ICELLNET: a transcriptome-based framework to dissect intercellular communication.
Prinz, M., Jung, S., and Priller, J. (2019). Microglia Biology: One Century of Evolving Concepts. Cell 179, 292-311.
Ramilowski, J.A., Goldberg, T., Harshbarger, J., Kloppmann, E., Lizio, M., Satagopam, V.P., Itoh, M., Kawaji, H., Carninci, P., Rost, B., et al. (2015). A draft network of ligand-receptor-mediated multicellular signalling in human. Nat Commun 6, 7866.
Richelle, A., Joshi, C., and Lewis, N.E. (2019). Assessing key decisions for transcriptomic data integration in biochemical networks. PLOS Computational Biology 15, e1007185-.
Song, Q., and Su, J. (2021). DSTG: deconvoluting spatial transcriptomics data through graph-based artificial intelligence. Briefings in Bioinformatics, 5.
Sun, D., Liu, Z., Li, T., Wu, Q., and Wang, C. (2022). STRIDE: accurately decomposing and integrating spatial transcriptomics using single-cell RNA sequencing. Nucleic Acids Research.
Yang, T., Alessandri-Haber, N., Fury, W., Schaner, M., Breese, R., LaCroix-Fralish, M., Kim, J., Adler, C., Macdonald, L.E., Atwal, G.S., et al. (2021). AdRoit is an accurate and robust method to infer complex transcriptome composition. Communications Biology 4, 1218.*

**Second round of review**

**Reviewer 2**

The authors have addressed all my concerns or provided reasonable clarifications. I don't have any comments for the revised manuscript.
